# Anchoring ALS Prognosis: Neurofilament Light Chain Outperforms Inflammatory, Metabolic, and CNS Barrier Biomarkers in the METABALS Cohort

**DOI:** 10.1007/s12035-026-05949-y

**Published:** 2026-05-28

**Authors:** Hugo Alarcan, Charlotte Veyrat-Durebex, Pierre-François Pradat, Julien Cassereau, Alain Destee, Philippe Couratier, William Camu, Jean-Philippe Neau, Marie-Céline Fleury-Lesaunier, Patrick Emond, Diane Dufour, Yara Al Ojaimi, Antoine Lefèvre, Patrick Vourc’h, Philippe Corcia, Christian R. Andres, Hélène Blasco

**Affiliations:** 1 Service de Biochimie Et Biologie Moléculaire, CHRU Tours, Tours, France; 2 Université de Tours, INSERM, Imaging Brain & Neuropsychiatry iBraiN U1253, 37032 Tours, France; 3 APHP, Département de Neurologie, Hôpital Pitié-Salpêtrière, Centre de Référence SLA, Paris, France; 4 Sorbonne Université, CNRS, INSERM, Laboratoire d’Imagerie Biomédicale, Paris, France; 5 Service de Neurologie, Centre de Référence Des Maladies Neurogénétiques, CHU d’Angers, Pôle JUPITER, Angers, France; 6 MITOVASC, UMR CNRS 6015-INSERM U1083, Equipe Mitolab, Université d’Angers, Angers, France; 7 Service de Neurologie Et Pathologie du Mouvement Clinique de Neurologie, CHU de Lille, Lille, France; 8 Centre de Référence SLA Et Autres Maladies du Neurone Moteur, CHU Dupuytren 1, Limoges, France; 9 INM, Université de Montpellier, INSERM, Montpellier, France; 10 Centre de Référence SLA, CHU de Montpellier, Montpellier, France; 11 Service de Neurologie, CHU La Milétrie, Hôpital Jean Bernard, Poitiers, France; 12 Service de Neurologie, CHU de Strasbourg, Strasbourg, France; 13 Service de Médecine Nucléaire in Vitro, CHRU de Tours, Tours, France; 14 Plateforme de Métabolomique Et d’Analyses Chimiques, Université de Tours, CHRU Tours, Inserm, US61 ASB Tours, France; 15 Service de Neurologie, CHRU de Tours, Tours, France

**Keywords:** Amyotrophic lateral sclerosis, Neurofilament light chain, Central nervous system, Metabolomic, Biomarkers

## Abstract

Amyotrophic lateral sclerosis (ALS) is a rapidly progressive and fatal neurodegenerative disorder with marked biological heterogeneity. Despite extensive research, reliable prognostic biomarkers remain limited, with neurofilament light chain (NfL) being the only marker increasingly implemented in clinical practice. The objective of this study is to assess and compare the prognostic value of NfL, circulating markers of central nervous system (CNS) barrier dysfunction, inflammatory mediators, kynurenine pathway metabolites, and global metabolomic profiles in patients with ALS. Seventy-two patients with ALS from the prospective multicenter METABALS cohort were included. Serum, cerebrospinal fluid (CSF), and urine samples were collected at diagnosis. NfL concentrations, markers of blood–brain and blood–spinal cord barrier permeability (albumin quotient, S100B, neuron-specific enolase [NSE]), 48 inflammatory mediators, kynurenine pathway metabolites, and untargeted metabolomic profiles were measured. Associations with clinical features, disease progression, and survival were investigated using univariate analyses and multivariate models. Serum and CSF NfL concentrations were strongly associated with ALS Functional Rating Scale–Revised scores, respiratory function, diagnostic delay, and survival. Higher serum NfL concentrations at diagnosis predicted shorter survival (ROC AUC = 0.86). In all multivariate and multi-block models, serum NfL was the only biomarker independently associated with survival. Markers of CNS barrier integrity, inflammatory mediators, and metabolomic signatures showed limited prognostic value but provided insights into metabolic remodeling and barrier dysfunction. In this integrated multi-omics study, serum NfL clearly outperformed inflammatory, metabolic, and CNS barrier markers as a prognostic biomarker in ALS, supporting its central role in clinical stratification while complementary biological markers highlighted several relevant pathophysiological mechanisms.

**Supplementary Information:**

The online version contains supplementary material available at 10.1007/s12035-026-05949-y.

## Introduction

Amyotrophic lateral sclerosis (ALS) is a relentlessly progressive neurodegenerative disorder characterized by the degeneration of upper and lower motor neurons [1]. It is the most frequent adult-onset motor neuron disease and is typically fatal within 3 to 5 years after symptom onset, most often due to respiratory failure [1]. Despite decades of intensive research, ALS pathogenesis remains incompletely understood and is thought to arise from the convergence of multiple molecular mechanisms, including glutamate-mediated excitotoxicity, oxidative stress, mitochondrial dysfunction, impaired RNA metabolism, protein aggregation, and neuroinflammation [2]. More than 30 genes have been implicated in hereditary forms of ALS, with pathogenic variants most commonly affecting *C9orf72*, *TARDBP*, *SOD1*, and *FUS*. Most cases remain sporadic, highlighting the role of non-genetic, systemic factors. This complexity parallels repeated clinical trial failures and the continued scarcity of reliable diagnostic and prognostic biomarkers, despite increasing use of neurofilament light chain (NfL) in practice [3].

Biomarker discovery is central in ALS research. Central nervous system (CNS) barrier impairment, including blood–brain barrier (BBB) and blood–spinal cord barrier (BSCB) dysfunction, occurs in ALS [4] and may contribute to pathogenesis [5]. The albumin quotient (QAlb) is the most used in vivo marker of barrier permeability and shows associations with survival, though mechanisms are unclear. Other circulating markers, such as S100β, neuron-specific enolase (NSE), and glial fibrillary acidic protein (GFAP), have been proposed, but their clinical relevance remains uncertain [6].

Beyond barrier dysfunction, inflammatory and metabolic pathways are increasingly studied. Neuroinflammation is a hallmark of ALS and may affect progression, but cytokine variability has produced inconsistent prognostic results [7]. Combining inflammatory mediators into multivariate signatures, rather than relying on single markers, may improve prognostic relevance [7]. Closely intertwined with inflammatory processes, the kynurenine (KYN) pathway (KP) of tryptophan (TRP) metabolism [8] has emerged as a potential contributor to ALS pathophysiology, with evidence of imbalance between neuroprotective and neurotoxic metabolites and associations with disease prognosis [9, 10]. In parallel, metabolomic profiling has gained momentum as a powerful, hypothesis-free approach to capture systemic metabolic remodeling and to provide mechanistic insights into ALS biology [11].

Against this background, the present study aimed to comprehensively evaluate the prognostic value of NfL, circulating markers of BBB impairment, inflammatory mediators, kynurenine pathway metabolites and metabolomic profiles in serum, cerebrospinal fluid (CSF), and urine within a well-characterized prospective ALS cohort (METABALS, ClinicalTrials.gov: NCT01962311). By integrating these complementary biological layers, this work also seeks to advance understanding of the complex pathophysiological processes underlying ALS beyond neuron-centric models.

## Material and Methods

### Study Population

This study used samples from the multicenter prospective METABALS cohort, designed to evaluate CSF, blood, and urine metabolites as early ALS biomarkers. Eighty-one patients with probable or definite ALS (Airlie House criteria [12]) aged 30–80 were included, providing informed consent and social security affiliation. Recruitment occurred at ALS centers in Tours, Poitiers, Angers, Paris, Lille, and Strasbourg (Feb 2013–Oct 2016). Exclusion criteria were prior riluzole treatment or participation in another study. Demographic and clinical data collected included age at onset, site of onset, disease duration, sex, and reference weight.

The following data were recorded at inclusion and at each follow-up visit (every 6 months for up to 36 months): vital status, El Escorial criteria classification [12], ALS Functional Rating Scale-Revised (ALSFRS-R) score, forced vital capacity (FVC), slow vital capacity (SVC), weight, and body mass index (BMI). Progression rate of ALSFRS-R at diagnosis (dALSFRS-R) was calculated as follows: (48 − ALSFRS-R)/disease progression (months). Variation in reference weight was determined using the following formula: (Weight at consultation − Reference weight)/Reference weight × 100.

The METABALS study was approved by the ethical committee of the Tours Hospital. All patients provided written informed consent. The study was registered on Clinicaltrial.gov under the registration number NC T01962311 in February 2013.

### Sample Collection and Processing

At inclusion, corresponding to the time of diagnosis, blood and CSF samples were collected by venipuncture and lumbar puncture, respectively, and urine samples were also obtained. Serum samples were prepared by centrifuging blood samples at 3000 g for 10 min at room temperature (RT). All samples were stored at −80 °C until further analyses.

For the present study, only patients for whom at least 2 aliquots of CSF and blood were available were included. NfL, markers of CNS barrier integrity, inflammatory mediators, KP, and metabolomic profiles were determined in serum and CSF. Metabolomic profiles were also assessed in urine.

### NfL Quantification

Serum and CSF NfL concentrations were quantified using the chemiluminescent enzyme immunoassays (CLEIA) Lumipulse® G NfL Blood and Lumipulse® G NfL CSF, respectively, on the Lumipulse® G600 II automated instrument (Fujirebio, Tokyo, Japan), according to the manufacturer’s instructions. The limit of detection (LoD) and limit of quantification (LoQ) for the Lumipulse® assay were determined to be 3.6 pg/mL and 6.1 pg/mL, respectively (according to the manufacturer’s). All factors of variability were low for this assay, with %CVs between 3.3 and 7.0% for the highest and lowest neat CSF samples.

### Measurement of Circulatory Markers of CNS Barriers Integrity

Serum and CSF albumin concentrations were determined using a COBAS® 6000 analyzer (Roche Diagnostics, Meylan, France) and an immunoturbidimetric assay, as routinely performed in our biochemistry laboratory. QAlb was calculated using the following formula: QAlb (%) = CSF albumin/serum albumin × 100.

The age-related upper reference limit was calculated as previously described: (4 + Age/15) × 10^−1^ [13]. Serum and CSF S100B and NSE concentrations were determined using electrochemiluminescence immunoassay (COBAS® 6000 analyzer (Roche Diagnostics Meylan, France)). The corresponding quotients (QS100B and QNSE) were calculated using the following formula: QS100B (%) = CSF S100B/serum S100B × 100 and QNSE (%) = CSF NSE/serum NSE × 100.

### Inflammatory Mediators

Concentrations of inflammatory mediators were determined by Luminex® technology with the Bio-Plex® Pro Human Cytokine Screening Panel, 48-Plex (#12007283) allowing the measurement of 48 cytokines and chemokines. Analyses were performed according to manufacturer’s instructions and samples were read on a Bio-Plex® 200 system (Bio-Rad Laboratories, Inc., Hercules, CA, USA).

### Kynurenine Pathway Analysis

For tryptophan metabolite analysis, 50 µL of serum or CSF was mixed with 100 µL internal standards and 300 µL methanol. Calibration standards underwent the same preparation. After 30 min incubation at −20 °C, samples were centrifuged (15,000*g*, 15 min, 4 °C). Supernatants (300 µL) were transferred to a 96-well plate, evaporated, and resuspended in 100 µL methanol/water (10:90); 5 µL was injected into an XEVO-TQ-XS LC–MS system with a Kinetex C18 XB column (1.7 µm × 150 mm × 2.1 mm, 55 °C). Two mobile phases were used (A: water + 0.5% formic acid; B: MeOH + 0.5% formic acid) at 0.4 mL/min. Gradient, Unispray® ion source, and fragmentation parameters were as previously described [14]. For each metabolite, a calibration curve was created by calculating the intensity ratio obtained between the metabolite and its internal standard. These calibration curves were then used to determine the concentrations of each metabolite in patient samples. IDO-1 enzyme activity was estimated by the KYN/TRP ratio [15], while other enzyme activities were reflected by metabolite ratios such as kynurenic acid (KYNA)/KYN, 3-hydroxykynurenine (3-HK)/KYN, quinolinic acid (QUIN)/KYNA, QUIN/KYN, and 3-HK/KYNA [16].

### Metabolomics Analyses

Metabolites were extracted by adding methanol to serum (50 µL), CSF (20 µL) or urine (100 µL, 1:10 dilution) and incubating at − 20 °C for 30 min. After centrifugation (3000 rpm, 4 °C, 30 min), 350 µL supernatant was evaporated at 40 °C and resuspended in 100 µL water/ACN (80/20); 5 µL were injected into a UPLC Ultimate WPS-3000 system coupled to a Q-Exactive mass spectrometer, operated in positive and negative ESI modes. Chromatography was performed on a Phenomenex Kinetex XB-C18 column (150 mm × 2.1 mm, 1.7 µm) at 40 °C, using a water/formic acid (0.%) − acetonitrile/formic acid (0.1%) gradient at 0.4 mL/min. A multistep gradient ran from 0.1% to 99.9% B over 20 min.

For increased coverage, a HILIC column (CORTECS UPLC HILIC 150 mm × 2.1 mm × 1.6 µm) was used. Samples were added to ACN, centrifuged, and 5 µL injected. Gradients used water + 10 mM NH4 formate + 0.5% formic acid (A) and ACN + 10 mM NH4 formate + 0.5% formic acid (B) at 0.4 mL/min over 22 min. Instrument stability was monitored using pooled QC samples analyzed at the start, every 10 injections, and end of the run; only metabolites with CV < 30% in QC were retained. Metabolites were identified using the MSML® library of 495 standards (IROA Technologies®). To identify the metabolites, several criteria were used as previously reported [17]. The signal intensity value was calculated using Xcalibur® 2.2 software (Thermo Fisher Scientific, San Jose, CA, USA).

### Statistical Analysis

CSF/serum ratios were calculated for each metabolite and inflammatory mediator (CSF analyte/serum analyte) and used as an additional matrix. Missing values were replaced by the analyte’s minimal signal divided by five [18]. Only analytes with ≥ 5 distinct values were retained. Patterns and outliers were explored by PCA; outliers were excluded.

Univariate analyses evaluated associations between biological features and diagnostic parameters (sex, onset site, age, ALSFRS-R, dALSFRS-R, weight, FVC, SVC, diagnostic delay) using Wilcoxon or Spearman tests. Associations with disease progression (variation in ALSFRS-R, weight, FVC over 1 year) were also assessed overall and by sex; only overall results are reported.

For survival analyses, patients were grouped by median disease duration. Demographic variables were compared between the two groups using Wilcoxon and chi-square tests for continuous and categorical variables, respectively. Univariate Wilcoxon tests identified candidate biomarkers, followed by multivariate sparse PLS-DA (sPLS-DA) models. Features were autoscaled and log10-transformed; models included biological features alone or combined with clinical features. A multi-block sPLS-DA was also performed (NfL, BBB integrity markers, cytokines, metabolites). Model quality was assessed via cross-validated prediction accuracy and permutation tests (100 permutations). ROC analysis evaluated key feature performance.

Associations between biological features and known progression markers (NfL, BBB markers) were explored using Spearman correlations and PLS models; final models retained features with VIP > 0.8. Correlations between BBB markers were also calculated. *P*-values were FDR-adjusted; significance threshold was *p* < 0.05. Analyses were performed in R (v2022.02.3) using MixOmics (multivariate), RVAideMemoire (permutations), and pROC (ROC).

## Results

### Demographics

Seventy-two patients with ALS for whom paired cerebrospinal fluid and serum samples (two aliquots each) were available were included in the present study. Demographic and clinical characteristics are summarized in Table 1. Overall, the median age at symptom onset was 61.7 years, with a median diagnostic delay of 13.3 months. Median disease duration from symptom onset to death was 40.9 months. We observed that 65.3% of patients were male (*n* = 47).

**Table 1:** Clinical characteristic of included patients

Variable	All patients (*N* = 72)	Short survival (*N* = 28)	Long survival (*N* = 37)	FDR
***Sex***				0.55
Male (*N*, %)	47 (65.3%)	16 (57.1%)	25 (67.6%)	
Female (*N*, %)	25 (34.7%)	12 (42.9%)	12 (32.4%)	
Age of onset (years)	61.7 (48.4–67.1)	64.2 (60.5–69.9)	59.7 (47.1–66.8)	
***Site of onset***				0.14
Spinal (*N*, %)	55 (76.4%)	18 (64.3%)	31 (83.8%)	
Bulbar (*N*, %)	17 (23.6%)	10 (35.7%)	6 (16.2%)	
***At diagnosis***				
ALSFRS-R	43 (39–44)	40 (37.5–43)	43 (39.8–45)	0.023
dALSFRS-R	0.44 (0.29–0.93)	1.11 (0.46–1.7)	0.36 (0.15–0.5)	< 0.001
Weight (kg)	69.5 (63–77)	65.7 (58–70.5)	72 (68–79)	0.014
Variation in reference weight (%)	− 3.0 (− 9.0–0.0)	− 4.4 (− 9.9– − 2.6)	− 2.0 (− 7.2–0.0)	0.13
FVC (%)	103.5 (87–119)	90.5 (80.5–107.3)	110 (98–122)	0.014
SVC (%)	107. (923–119)	100 (74.8–108.5)	108 (100–119)	0.013
Diagnostic delay (months)	13.3 (7.4–18.0)	8.4 (5.0–12.1)	17.0 (13.2–25.0)	< 0.001
***Variation after a year (%)***				
ALSFRS-R	− 20.2 (− 37.4– − 10.6)	− 36.8 (− 49.2– − 15.7)	− 14.6 (− 31– − 2.9)	0.015
Weight	− 1.6 (− 9.6–1.9)	− 8.8 (− 13.0– − 1.6)	0.0 (− 4.3–2.4)	0.023
FVC	− 10.6 (− 34.9– − 7.6)	− 41.7 (− 43.9– − 21.6)	− 8.6 (− 10.7– − 6.2)	0.014
SVC	− 9.5 (− 28.9– − 4.1)	− 36.1 (− 39.4– − 29.2)	− 5.5 (− 15.3– − 2.8)	0.007
***Disease duration before death (months***)	40.9 (27.0–65.8)	27.0 (20.5–31.4)	70.1 (54.4–93.2)	< 0.001

Continuous variables are represented as median (interquartile range). Information about age of onset, ALSFRS-R at diagnostic, dALSFRS-R, FVC at diagnosis, SVC at diagnosis, % in regard to normal values, diagnostic delay, variation in ALSFRS-R, weight, FVC, SVC over a year, and about death was missing for 1.4, 2.8, 4.2, 11.1, 30.6, 1.4, 41.7, 29.2, 73.6, 59.7, and 23.6% of patients, respectively. *ALSFRS*-*R*, ALS functional rating scale-revised; *dALSFRS*-*R*, progression rate of ALSFRS-R; *FVC*, forced vital capacity; *SVC*, slow vital capacity; *FDR*, false discovery rate

### Determination of Biological Markers

NfL concentrations were determined in all patients except for one CSF sample (insufficient volume), with a median (Interquartile (IQR) range) value of 3999 (2448–6498) and 78.89 (43.10–109.52) pg/mL in CSF and serum, respectively. Serum and CSF concentrations of NfL were correlated (*R*^*2*^ = 0.47, *p* < 0.0001) (Figure S1A). The distribution of circulatory markers of BBB integrity is available in supplementary Table S1. CSF and serum metabolomic profiles were determined in all patients, while for urine, samples were not available for two of the patients. We found a total of 236, 108, and 233 metabolites in serum, CSF, and urine, respectively (Table S2). As revealed by the Venn diagram (Figure S1B), 67 metabolites were common to all matrices and only 4 metabolites were specifically found in the CSF. PCA identified four outliers in the serum metabolomic profiles and one outlier in the CSF metabolomic profiles, which were excluded from all subsequent statistical analyses involving metabolites. Inflammatory cytokines were assessed in all patients for both serum and CSF samples (Table S3). GM-CSF and IFN-a2 in the CSF and GM-CSF, IL-5, IL-12 p40, IL-15, and VEGF in the serum were detected in very few patients and were therefore removed from subsequent analyses. Similarly, for the KP, tryptophol and melatonin were removed from serum and CSF analyses, and serotonin, tryptamine, indole-3-sulfate, N-acetylserotonin, xanthurenic acid, indole-3-lactic acid, and indole-3-aldehyde were removed from CSF analyses as they were detected in very few patients. Distributions of KP intermediates are available in supplementary Table S3B.

### Correlation Between Circulatory Markers and Clinical Parameters at Inclusion

#### Gender

**Fig. 1:**
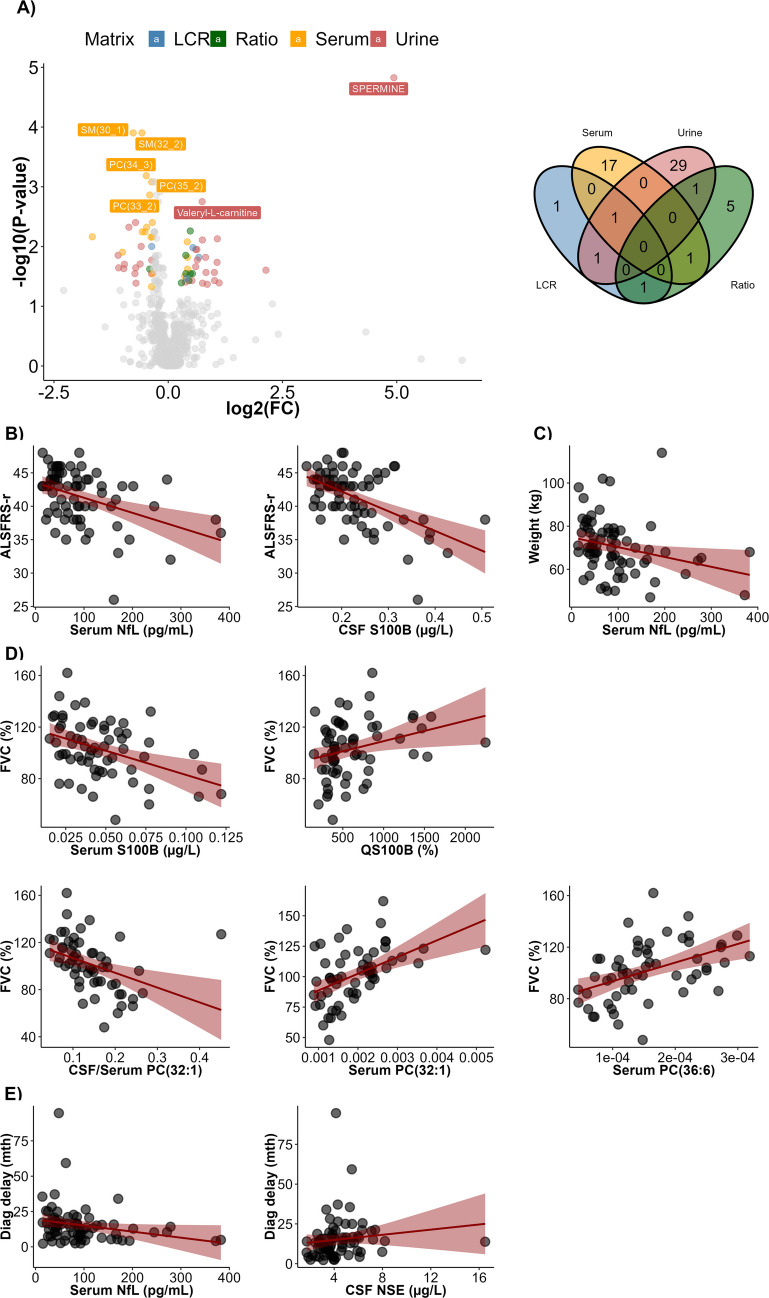
Correlation between biological features and clinical parameters of the disease at diagnosis. **A** On the left, volcano plot representing the different features between males and females. FC between male and female subjects is represented by the *x*-axis (log2 scale) and the adjusted *p*-value by the *y*-axis (negative log10 scale). On the right, Venn diagram representing the common and matrix-specific metabolites that are significantly different between males and females. Valeryl-L-carnitine is common to serum, CSF, and urine. Ethanolamine phosphate is common to CSF and urine. Urate is common to CSF and CSF/serum ratio. PC(30:0) is common to serum and CSF/serum ratio. SM(34:1) is common to CSF/serum ratio and urine. **B** Correlation plots between ALSFRS-R at diagnosis and serum NfL or CSF S100B. **C** Correlation plot between weight at diagnosis and serum NfL. **D** Correlation plots between FVC at diagnosis and serum S100B, QS100B, CSF/Serum and serum PC(32:1), or serum PC(36:6). **E** Correlation plots between diagnostic delay and serum NfL or CSF NSE. The red band represents the 95% confidence concentration interval

We found no difference between males and females for serum and CSF NfL, nor for markers of the BBB integrity and inflammatory mediators after adjustment of the *p*-values. To note, QAlb was not different between males and females even when considering raw *p*-values (Figure S2A). When looking at the age-related upper reference limit, 13 patients (18%) had an elevated QAlb: 3 females (12%) and 10 males (21%) (Figure S2B). As for metabolites, when considering a threshold of 0.05 for FDR and 0.8 or 1.2 for fold change (FC), 63 features were significantly different between males and females, with only 5 metabolites common to at least 2 matrices (Fig. 1A). Concerning the KP intermediates, picolinic acid (FC = 1.74, FDR = 0.001), TRP (FC = 1.14, FDR = 0.01), and xanthurenic acid (FC = 1.46, FDR = 0.03) were significantly higher in males.

#### Onset Site and Age of Onset

After adjustment for multiple comparisons, no biological features differed significantly between spinal and bulbar forms of ALS, either in the overall cohort or after sex stratification. Age at onset was not correlated with NfL concentrations (CSF and serum) or markers of BBB integrity. In contrast, age at onset was significantly associated with nine inflammatory mediators, ten metabolites, and 14 kynurenine pathway intermediates in the overall population (see Table S4 for detailed results and sex-specific associations).

#### ALSFRS-R and dALSFRS-R Scores

Serum and CSF NfL were significantly correlated with ALSFRS-R score at diagnosis (*r *= −0.40, FDR = 0.001 and *r* = −0.37, FDR = 0.02, respectively) (Fig. 1B). To note, NfL concentrations were particularly associated with question 1 (speech) and 3 (swallowing) of the score. We also found a negative correlation between the ALSFRS-R score and CSF S100B (*r* = −0.44, FDR = 0.0015) (Fig. 1B). CSF S100B correlated particularly to question 5 (cutting food),7 (turning in bed), 8 (walking), 9 (climbing stair), and 10 (dyspnea). No inflammatory mediators, metabolites, or KP intermediates were associated with the score in the overall population (See Table S5 for gender-specific associations). Concerning dALSFRS-R, only serum and CSF NfL were significantly associated (data not shown).

#### Weight and Variation of Reference Weight

Weight at diagnosis was negatively correlated with serum NfL concentrations in the overall population (*r* = − 0.38, FDR = 0.0018; Fig. 1C). Thirteen metabolites and four KP intermediates were significantly associated with weight at diagnosis, whereas inflammatory mediators and markers of BBB integrity showed no significant correlations (see Table S6 for detailed and sex-stratified results). No biological feature was associated with changes relative to reference weight in the overall cohort (see Table S7 for detailed and sex-specific analyses).

#### Forced Vital Capacity and Slow Vital Capacity

Forced vital capacity (FVC) at inclusion was significantly associated with markers of BBB-related astroglial signaling, showing a negative correlation with serum S100B (*r* = − 0.37, false discovery rate FDR = 0.025) and a positive correlation with the CSF-to-serum S100B ratio (QS100B; *r* = 0.34, FDR = 0.026) (Fig. 1D). In addition, three lipid metabolites were associated with FVC, including CSF/serum and serum PC(32:1) (*r* = − 0.59, FDR < 0.001 and* r* = 0.55, FDR = 0.003, respectively), as well as serum PC(36:6) (*r* = 0.53, FDR = 0.005). No significant associations were observed with inflammatory mediators or kynurenine pathway intermediates (see gender-specific analyses in Table S8). No biological features were significantly associated with slow vital capacity (SVC).

#### Diagnostic Delay

The only features associated with diagnostic delay were serum and CSF NfL (*r* = −0.31; FDR = 0.015, and *r* = −0.27, FDR = 0.024, respectively), and CSF NSE (*r *= 0.34, FDR = 0.033) (Fig. 1E) in the overall population.

### Correlation Between Biological Features and Variation of Clinical Parameters Over a Year

Serum and CSF NfL were negatively correlated with the variation of ALSFRS-R score at 1 year in overall population (*r* = −0.5, FDR = 0.002 and *r *= −0.49, FDR = 0.003, respectively). Other biological features were not associated with this variable (see Table S9 for gender-specific associations). Serum and CSF NfL were also the only features associated with the variation of FVC over a year in the overall population (*r* = −0.61, FDR = 0.01, and *r* = −0.42, FDR = 0.04 for serum and CSF, respectively) (see Table S10 for gender-specific associations). No features were associated with the variation of weight at 1 year.

### Association Between Biological Features and Survival

We divided the cohort into two groups according to the median disease duration before death (short and long survival). Due to the limited size of our cohort, analyses were performed on the overall population within each group. Differences concerning clinical features can be found in Table 1. Briefly, significant differences were observed for ALSFRS-R, body weight, FVC at diagnosis, as well as for their annual variation, dALSFRS-R, and diagnostic delay.

**Fig. 2:**
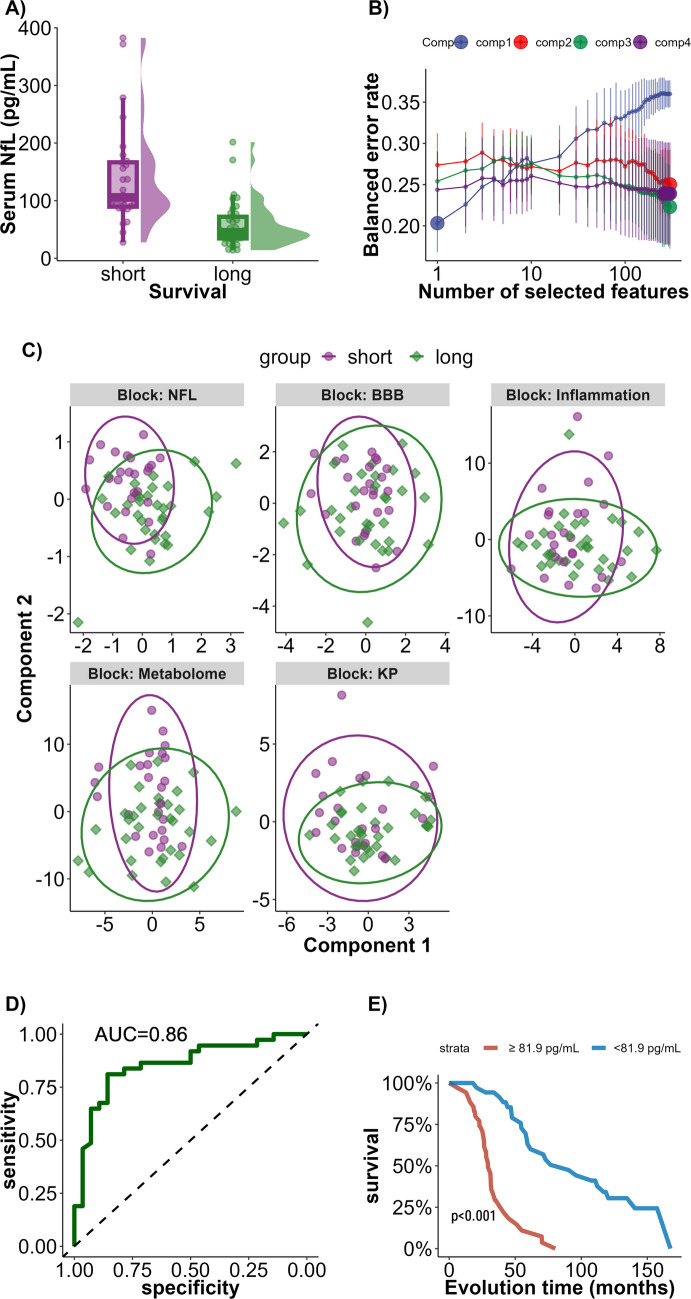
Association between biological features and survival. **A** Raincloud plots of serum NfL concentrations between short and long survival groups. **B** Results from the tuning of the sPLS-DA model built using biological features. Each line represents the number of components tested. *Y*-axis represents the balanced error rate (BER) of the survival group prediction and *x*-axis the number of features selected within each component. Data are represented as mean ± SD of the BER. Large points represent the number of selected features showing the best performances for each number of components. We can see that the minimal BER is obtained for one feature only in one component: serum NfL. **C** Score plots of the sPLS-DA model built using each dataset of biological variables as independent blocks. **D** ROC curve of the model including serum NfL alone to predict the median survival of ALS patients. **E** Kaplan–Meier curve with two groups set up according to the best threshold of serum NfL identified in the ROC analysis. AUC, area under the curve; BBB, blood–brain barrier; KP, kynurenine pathway

Concerning univariate analysis, serum NfL and CSF NfL were higher in patients with shorter survival (FC = 2.42 and 1.58, respectively, FDR < 0.001 for both tests) (example of serum NfL in Fig. 2A). No other features were significantly different between the two groups.

We built an sPLS-DA model to predict survival groups using biological and selected clinical features. Optimal performance relied on a single feature: serum NfL. A biological-only model gave similar results (Fig. 2B; average error rate 21%, significant by permutation).

A multi-block sPLS-DA including NfL, BBB markers, cytokines, and metabolites selected multiple features per block, but serum NfL alone outperformed the full model (21% vs 34% error), with clearer group separation in the NfL block (Fig. 2C).

ROC curve analysis for serum NfL showed an AUC of 0.86, with a threshold of 81.9 pg/mL identified to have a specificity and sensitivity of 85.7 and 81.1%, respectively (Fig. 2D). Kaplan–Meier survival analysis based on this threshold confirmed a shorter survival for patients with serum NfL concentrations above 81.9 pg/mL (Fig. 2E).

### Biological Features Associated with NfL

**Fig. 3:**
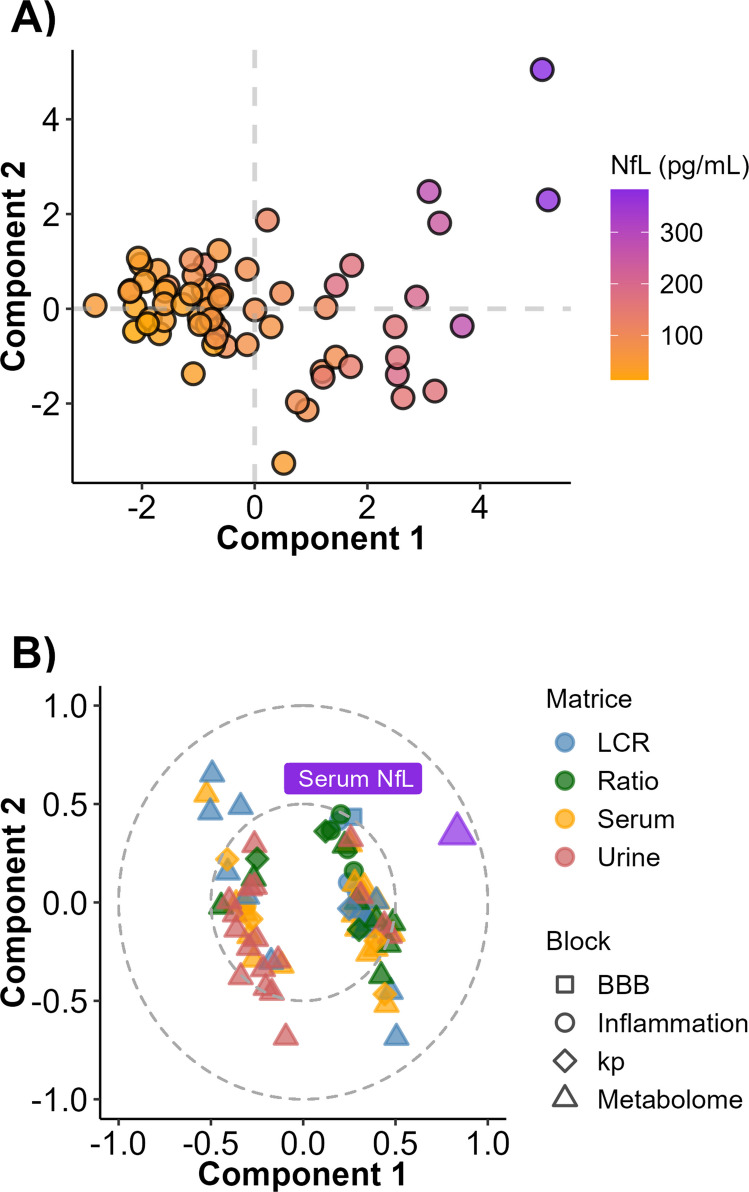
Interplay between the biological features. **A** Scores plot of the PLS model built to explain NfL with other biological features. **B** Loadings plot of the PLS model built to explain NfL with other biological features

In univariate analyses, no features were significantly associated with serum NfL after FDR adjustment of *p*-value in the overall population. The multivariate models showed similar and modest performances (*R*^*2*^ around 0.47 and *Q*^*2*^ around 0.47) in the overall population. Features included in the model can be found in Table S11. Score and loading plots of the PLS model are represented for the overall population in Fig. 3A and B.

## Discussion

### Serum Neurofilament Light Chain as the Central Prognostic Biomarker

In this prospective, multicentric cohort of patients with ALS, we performed an integrated evaluation of NfL, circulating markers of blood–brain barrier (BBB) integrity, inflammatory mediators, and metabolomic profiles. Although several biological features were associated with clinical parameters at diagnosis, our results consistently demonstrate that serum NfL clearly outperforms all other markers as a prognostic biomarker. Serum and CSF NfL showed the strongest and most consistent associations with key clinical variables, including ALSFRS-R score, weight at diagnosis, and diagnostic delay, and higher concentrations were robustly associated with shorter survival. Importantly, serum NfL was the only biomarker retained in multivariate prognostic models.

We identified a serum NfL threshold of 81.9 pg/mL at diagnosis that optimally discriminated patients according to median disease duration (40.9 months). This value is consistent with previously reported prognostic cutoffs around 80–85 pg/mL obtained using Simoa® technology [19, 20]. In contrast, several independent cohorts have reported higher serum NfL thresholds, typically in the 90–100 pg/mL range [20–22], for prognostic or diagnostic stratification across different analytical platforms. Importantly, a prognostic cutoff of approximately 95 pg/mL has been identified by Mondesert et al*.* [20] using Lumipulse® technology. In this study, Mondesert et al. conducted a French multicenter study evaluating the analytical performance of four sNfL immunoassays. They found that the Lumipulse assay showed consistent inter-laboratory reproducibility (CVs 2.0–16.9%) but noted significant batch-to-batch and inter-center variability (*p* < 0.001), particularly at low concentrations (10 pg/mL). Although all assays were highly correlated (*p* < 0.001), systematic biases were observed. This underscores the need for platform-specific reference ranges and harmonization efforts. The slightly lower prognostic threshold identified in our study (81.9 pg/mL) likely reflects differences in cohort composition, timing of sampling at diagnosis, and assay-specific calibration, while remaining fully consistent with the same biological continuum linking higher NfL concentrations to more aggressive disease. This convergence across studies reinforces the biological robustness of NfL as a marker of neuronal injury [23]. Nevertheless, inter-assay variability remains a major barrier to widespread clinical implementation, underscoring the need for assay-specific thresholds and harmonized outcome definitions [24]. To our knowledge, this study is among the first to characterize the prognostic performance of serum and cerebrospinal fluid NfL measured using Lumipulse® technology and our proposed cutoff is specific to the Lumipulse platform and should not be extrapolated to other methods (e.g., Simoa or ELISA) without prior validation.

NfL concentrations are influenced by age and disease stage. Disanto et al. demonstrated a 2.2% annual increase in serum NfL levels with age [25], while Khalil et al. reported a similar trend of 2–3% per year after age 50 [26]. Additionally, Benatar et al. highlighted stable NfL levels across different stages of neurodegenerative diseases [27]. These findings emphasize the importance of adjusting cutoffs based on patient demographics and clinical characteristics.

Beyond its clinical relevance, the biological mechanisms underlying NfL release in ALS remain incompletely understood. While NfL is a sensitive indicator of axonal damage [23], experimental data suggest that disease-specific processes involving TDP-43 or SOD1 may modulate neurofilament mRNA stability and release [28]. In our cohort, NfL concentrations were not correlated with inflammatory mediators, kynurenine pathway intermediates, or metabolomic features, suggesting an apparent independence at baseline between neuronal injury, as reflected by NfL concentrations, and systemic inflammatory or metabolic alterations. Longitudinal and stage-stratified analyses may reveal temporal coupling between these processes, which cannot be captured in a cross-sectional framework. This perspective aligns with emerging models of ALS pathophysiology integrating sequential or parallel processes.

Our findings align with and extend the current ALS biomarker landscape. Across cohorts and trials [29], NfL is the most robust biomarker of axonal injury, strongly associated with disease progression and survival, and increasingly used as a secondary or surrogate endpoint [30]. Our multi-omics analysis confirms that serum NfL provides superior prognostic discrimination compared with markers of BBB integrity, inflammation, kynurenine pathway activity, or metabolomic profiles. While these domains capture complementary ALS pathophysiology, none added independent prognostic value beyond NfL. Several studies have shown that NfL levels in ALS tend to rise early and then remain relatively stable over the course of disease progression in the absence of disease-modifying treatment [27]. Accordingly, baseline NfL levels can be considered a robust proxy of disease intensity and prognostic stratification while longitudinal changes in NfL may become particularly informative in treated populations or clinical trial settings, where deviations from this relative stability could reflect therapeutic response.

Our study further defines a clinically relevant prognostic threshold using Lumipulse®, supporting assay harmonization. The lack of correlation between NfL and inflammatory or metabolic signatures suggests NfL reflects a distinct, upstream aspect of ALS biology, complementing multi-omics insights into systemic and mechanistic contributors. Overall, these results reinforce serum NfL as the key prognostic biomarker, with metabolomic and inflammatory readouts serving primarily as tools for biological insight and therapeutic targeting.

### Limited Prognostic Value but Important Mechanistic Insights from Inflammation and Metabolomics

Neuroinflammation is widely recognized as a core component of ALS pathophysiology [1, 31]. Yet its contribution to disease progression remains debated [32]. In our cohort, inflammatory mediators in serum and CSF showed limited associations with clinical parameters or survival, consistent with previous reports [7, 33]. The main signal observed was a positive association between several inflammatory mediators and age at symptom onset in males, consistent with age-related increases in inflammatory activity and the sex-related differences described between male and female for this concept of “inflammaging” [34]. In females, only GRO-α decreased with aging, in line with postmenopausal changes reported in healthy populations [35].

These findings align with the concept that inflammatory responses in ALS are dynamic, with an early anti-inflammatory phase followed by later pro-inflammatory activation [36], which likely contributes to the heterogeneous and sometimes contradictory results reported across studies [7, 32]. Given their marked intra-individual variability [37] and dependence on disease stage and sampling timing, inflammatory mediators are unlikely to serve as robust standalone prognostic biomarkers in ALS. However, they remain highly informative for mechanistic exploration.

Similarly, only a limited number of metabolites were associated with clinical parameters in our study, often in sex-specific subgroups. Despite the strong potential of metabolomics to elucidate disease pathophysiology and inform therapeutic strategies, high biological variability and substantial inter-laboratory heterogeneity continue to limit its utility for robust biomarker discovery [14]. Nevertheless, converging evidence supports a role for metabolic remodeling in ALS across multiple biological matrices, including hypercatabolic signatures and mitochondrial dysfunction, alterations in amino acid, fatty acyl and purine metabolism, and even platelet-based metabolomic profiles, all consistent with systemic metabolic dysregulation accompanying neurodegeneration [38–40]. We identified metabolic alterations across serum, CSF, and urine; however, specific metabolites did not converge on a consensus signature. Thus, these omics-driven findings provide mechanistic insight rather than definitive clinical biomarkers.

We observed significant associations between body weight at diagnosis and several kynurenine pathway (KP) markers, including tryptophan, kynurenic acid, its ratio, and xanthurenic acid, suggesting coordinated modulation of substrate availability and downstream metabolism. Previous CSF studies reported minimal quantitative alterations of KP metabolites in early ALS, though subtle shifts toward potentially neurotoxic profiles were noted, with limited diagnostic value [41]. Our results extend these findings, showing that systemic KP markers in serum correlate with body weight, a key metabolic and prognostic feature, indicating that KP dysregulation may be more detectable peripherally than centrally in early disease. This aligns with comparative studies showing modest central KP changes but more pronounced serum alterations (e.g., reduced HK/XA ratios), contrasting with FTD or early-onset Alzheimer’s disease [42]. While KP activation is usually linked to neuroinflammation, associations with nutritional parameters are rarely explored. These findings highlight a novel link between body weight and KP activity, supporting peripheral KP markers as accessible and biologically meaningful biomarkers in ALS [43].

### Blood–Brain Barrier Dysfunction as a Mechanistic Component of ALS

BBB impairment has emerged as a potentially important contributor to ALS pathophysiology [5]. Recently, neuropathological and transcriptomic analyses revealed profound alterations of the choroid plexus and blood–CSF barrier, supporting an active role of barrier dysfunction in ALS rather than a purely secondary phenomenon [44]. While previous studies, including our own, reported associations between elevated QAlb and worse clinical outcomes [45, 46], we did not replicate these findings in the present cohort. This discrepancy likely reflects limited statistical power, cohort-specific differences, and a lower proportion of patients with elevated QAlb values. Importantly, circulating markers of BBB integrity were only weakly correlated with each other, underscoring the complexity of barrier alterations.

S100B, a calcium-binding protein primarily expressed by astrocytes, showed limited diagnostic and prognostic value, consistent with prior reports [47, 48]. Its poor correlation with other BBB markers suggests that extracerebral sources and alternative clearance pathways, including glymphatic transport or RAGE-mediated mechanisms, substantially contribute to circulating S100B concentrations [48, 49].

Neuron-specific enolase (NSE), although less extensively studied in ALS [50], showed associations with diagnostic delay and disease duration in sex-specific analyses.

## Strengths and Limitations

A major strength of the METABALS study is the depth of biological data generated through a standardized, prospective, multicenter design, enabling well-timed sample collection and detailed clinical characterization. This allowed comprehensive phenotyping integrating circulating biomarkers, inflammatory mediators, and multiple omics layers, supported by advanced analytics. Such a harmonized dataset enables exploration of molecular interactions and mechanistic signatures beyond single-biomarker approaches. Limitations include modest sample size, absence of genetic stratification, and prolonged sample storage. Future studies integrating genetically defined subgroups (e.g., *SOD1*, *C9orf72*, *TARDBP*, *FUS*) in larger cohorts may reveal subgroup-specific biomarker signatures and help refine prognostic models. Even though our primary objective lies in the head-to-head comparison of multiple biomarker classes within a single, prospectively collected cohort under standardized conditions, and the fact that the prognostic value of serum NfL is consistently supported across independent cohorts in the literature, the study would have been strengthened by external validation.

## Perspectives of Integrating Multi-Omics Approaches

Recent experimental and multi-omics studies reinforce that ALS is a systemic, heterogeneous disorder shaped by metabolic, lipid, and environmental factors. Longitudinal microbiome–metabolome analyses show strong associations between gut microbial composition and plasma lipid metabolism, highlighting microbiome–lipid interactions in disease mechanisms [51]. Environmental influences on lipid homeostasis, including diet and exposures, further exacerbate neurodegeneration. Integrative metallomic–metabolomic studies indicate that metal dyshomeostasis interacts with lipid and steroid metabolisms to promote oxidative stress and neuronal dysfunction [52]. Multi-omics approaches are better suited for mechanistic understanding and patient stratification than for predicting diagnosis or prognosis. Consistently, our integrated analysis of serum, CSF, and urine captures systemic metabolic and barrier remodeling in ALS, with substantial inter-individual variability. Although these signatures do not yield biomarkers beyond NfL, this reflects inherent limitations of omics and underscores the need to integrate internal and external environmental factors—metabolism, microbiota, nutrition, and exposome—to address disease pathophysiology and heterogeneity.

## Conclusion

In conclusion, our study confirms that serum NfL is the most robust prognostic biomarker in ALS, clearly surpassing inflammatory mediators, metabolomic profiles, and circulating markers of BBB integrity. Together, these findings support a model in which neuronal injury, metabolic stress, barrier alterations, and environmental influences interact to shape ALS progression, reinforcing the value of integrative, multi-omics and exposome-oriented approaches alongside established clinical biomarkers.

## Supplementary Information

Below is the link to the electronic supplementary material.

ESM1 (DOCX 419 KB)

ESM2 (DOCX 60.7 KB)

## Data Availability

The datasets generated and analyzed during the current study are available from the corresponding author on reasonable request.

## Declarations

### Approvals, Registrations, and Patient Consents

The METABALS study was approved by the ethical committee of the Tours Hospital. All patients provided written informed consent. The study was registered on Clinicaltrial.gov under the registration number NC T01962311 in February 2013.
