# Supplementary material for: Anchoring ALS Prognosis: Neurofilament Light Chain Outperforms Inflammatory, Metabolic, and CNS Barrier Biomarkers in the METABALS Cohort
Source: Mol Neurobiol. 2026 May 28;63(1):657. doi: 10.1007/s12035-026-05949-y (PMC13219083; doi:10.1007/s12035-026-05949-y)
Supplement: Supplementary file 1 — (DOCX 419 KB) [file 12035_2026_5949_MOESM1_ESM.docx]

**Molecular Neurobiology**

**Anchoring ALS prognosis: neurofilament light chain outperforms inflammatory, metabolic and CNS barrier biomarkers in the METABALS cohort**

Hugo Alarcan^1,2^, Charlotte Veyrat-Durebex^1,2^, Pierre-François Pradat^3,4^, Julien Cassereau^5,6^, Alain Destee^7^, Philippe Couratier^8^, William Camu^9,10^, Jean-Philippe Neau^11^, Marie-Céline Fleury-Lesaunier^12^, Patrick Emond^2,13,14^, Diane Dufour^2,13^, Yara Al Ojaimi^2^, Antoine Lefèvre^2,14^, Patrick Vourc’h^1,2^, Philippe Corcia^2,15^, Christian R. Andres^1,2*^, Hélène Blasco^1,2*^

**Affiliations**

1 Service de Biochimie et Biologie Moléculaire, CHRU Tours, Tours, France

2 Université de Tours, INSERM, Imaging Brain & Neuropsychiatry iBraiN U1253, 37032, Tours, France

3 APHP, Département de Neurologie, Hôpital Pitié-Salpêtrière, Centre de référence SLA, Paris, France.

4 Sorbonne Université, CNRS, INSERM, Laboratoire d'Imagerie Biomédicale, Paris, France.

5 Service de Neurologie, Pôle JUPITER, Centre de Référence des Maladies Neurogénétiques, CHU d’Angers, France

6-MITOVASC UMR CNRS 6015-INSERM U1083, Equipe Mitolab, Université d’Angers

7 Service de Neurologie et Pathologie du Mouvement Clinique de Neurologie, CHU de Lille, Lille, France

8 Centre de Référence SLA et autres maladies du neurone moteur, CHU Dupuytren 1, Limoges, France

9 INM, Université de Montpellier, INSERM, Montpellier, France

10 Centre de Référence SLA, CHU de Montpellier, Montpellier, France

11 Service de Neurologie, CHU la Milétrie, Hôpital Jean Bernard, Poitiers, France.

12 Service de Neurologie, CHU de Strasbourg, Strasbourg, France

13 Service de Médecine nucléaire in vitro, CHRU de Tours, Tours, France

14 Plateforme de Métabolomique et d'Analyses Chimiques, US61 ASB, Université de Tours, CHRU Tours, Inserm, Tours, France

15 Service de Neurologie, CHRU de Tours, Tours, France

* These authors contributed equally

Corresponding author, Hélène Blasco, helene.blasco@univ-tours.fr


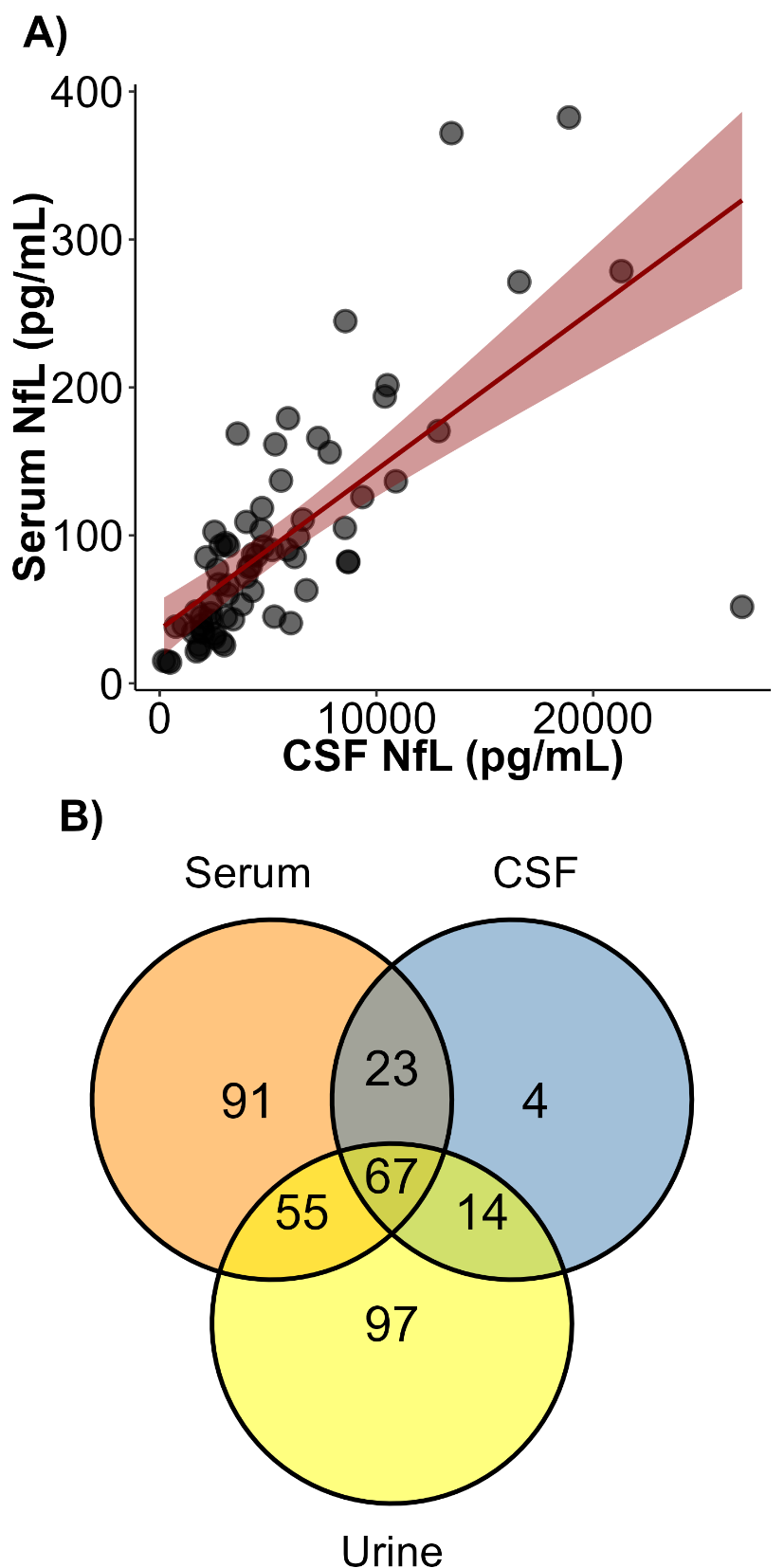


**Supplementary Figure S1: Determination of biological markers.** A) Correlation between serum and CSF NfL in overall population. Red band represents the 95% confidence level interval. B) Venn diagram representing the number of common and specific metabolites found in serum, CSF and urine.


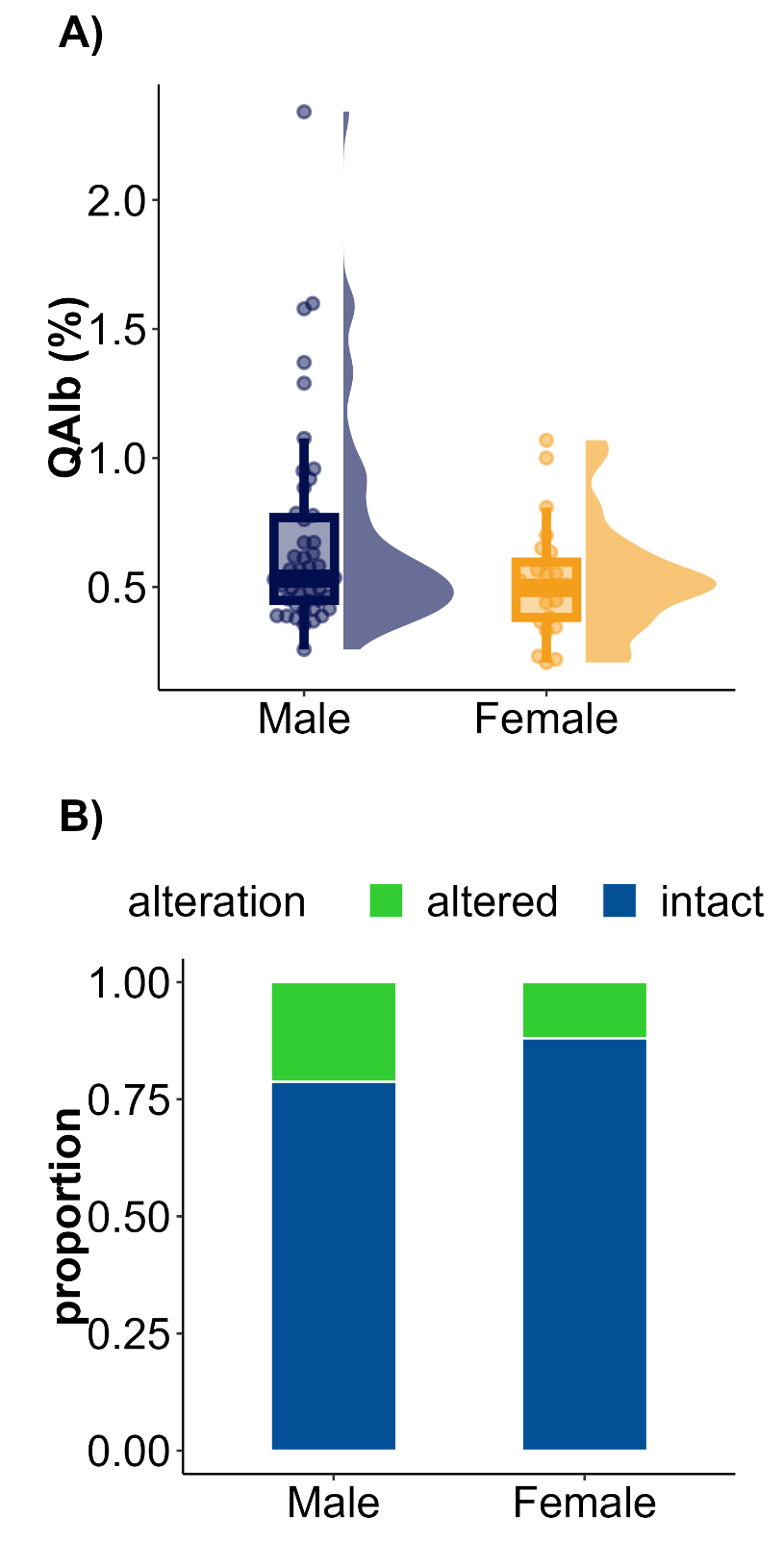


**Supplementary Figure S2: Association between QAlb and sex.** A) Raincloud plots of QAlb values between male and female ALS patients. B) Proportion of males and females with an alteration in BBB according to the age-limited reference value: (4 + age/15) × 10−1.


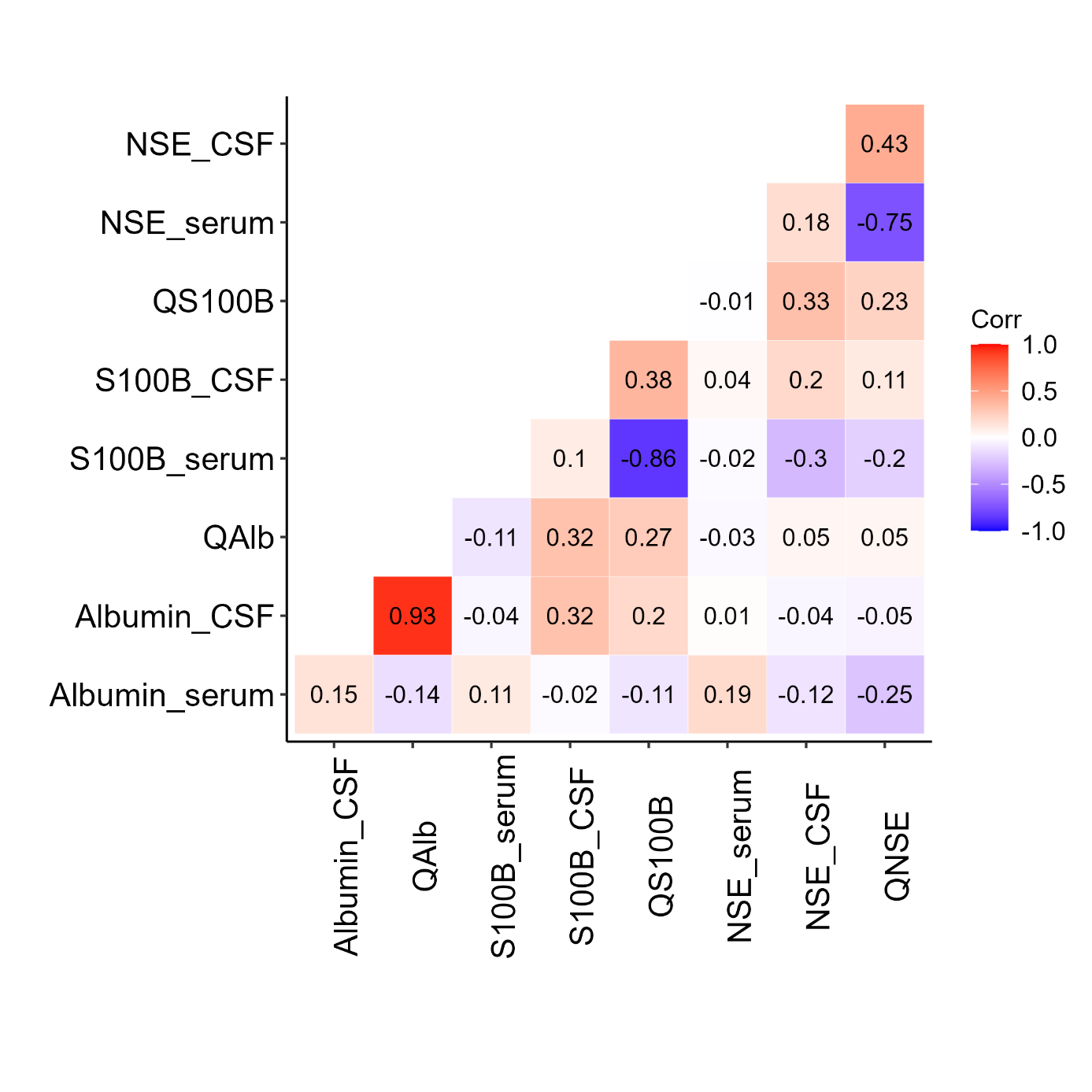


**Supplementary Figure S3: Correlation matrix of the circulatory markers of BBB integrity.** Spearman coefficient correlation was used. Blue and red boxes represent negative and positive correlation, respectively
