# Supplementary material for: Anchoring ALS Prognosis: Neurofilament Light Chain Outperforms Inflammatory, Metabolic, and CNS Barrier Biomarkers in the METABALS Cohort
Source: Mol Neurobiol. 2026 May 28;63(1):657. doi: 10.1007/s12035-026-05949-y (PMC13219083; doi:10.1007/s12035-026-05949-y)
Supplement: Supplementary file 2 — (DOCX 60.7 KB) [file 12035_2026_5949_MOESM2_ESM.docx]

**Molecular Neurobiology**

**Anchoring ALS prognosis: neurofilament light chain outperforms inflammatory, metabolic and CNS barrier biomarkers in the METABALS cohort**

Hugo Alarcan^1,2^, Charlotte Veyrat-Durebex^1,2^, Pierre-François Pradat^3,4^, Julien Cassereau^5,6^, Alain Destee^7^, Philippe Couratier^8^, William Camu^9,10^, Jean-Philippe Neau^11^, Marie-Céline Fleury-Lesaunier^12^, Patrick Emond^2,13,14^, Diane Dufour^2,13^, Yara Al Ojaimi^2^, Antoine Lefèvre^2,14^, Patrick Vourc’h^1,2^, Philippe Corcia^2,15^, Christian R. Andres^1,2*^, Hélène Blasco^1,2*^

**Affiliations**

1 Service de Biochimie et Biologie Moléculaire, CHRU Tours, Tours, France

2 Université de Tours, INSERM, Imaging Brain & Neuropsychiatry iBraiN U1253, 37032, Tours, France

3 APHP, Département de Neurologie, Hôpital Pitié-Salpêtrière, Centre de référence SLA, Paris, France.

4 Sorbonne Université, CNRS, INSERM, Laboratoire d'Imagerie Biomédicale, Paris, France.

5 Service de Neurologie, Pôle JUPITER, Centre de Référence des Maladies Neurogénétiques, CHU d’Angers, France

6-MITOVASC UMR CNRS 6015-INSERM U1083, Equipe Mitolab, Université d’Angers

7 Service de Neurologie et Pathologie du Mouvement Clinique de Neurologie, CHU de Lille, Lille, France

8 Centre de Référence SLA et autres maladies du neurone moteur, CHU Dupuytren 1, Limoges, France

9 INM, Université de Montpellier, INSERM, Montpellier, France

10 Centre de Référence SLA, CHU de Montpellier, Montpellier, France

11 Service de Neurologie, CHU la Milétrie, Hôpital Jean Bernard, Poitiers, France.

12 Service de Neurologie, CHU de Strasbourg, Strasbourg, France

13 Service de Médecine nucléaire in vitro, CHRU de Tours, Tours, France

14 Plateforme de Métabolomique et d'Analyses Chimiques, US61 ASB, Université de Tours, CHRU Tours, Inserm, Tours, France

15 Service de Neurologie, CHRU de Tours, Tours, France

* These authors contributed equally

Corresponding author, Hélène Blasco, helene.blasco@univ-tours.fr

**Supplementary Table S1: distribution of circulatory markers of the BBB integrity**

| **Variable** | **Median, IQR** |
| --- | --- |
| Serum Albumin (g/L) | 45.5 (41.58- 47.70) |
| CSF Albumin (g/L) | 0.24 (0.20- 0.30) |
| QAlb (%) | 0.53 (0.44- 0.67) |
| Serum S100B (µg/L) | 0.04 (0.03- 0.06) |
| CSF S100B (µg/L) | 0.20 (0.17- 0.26) |
| QS100 (%) | 493 (366.8- 763) |
| Serum NSE (µg/L) | 15.16 (10.24- 19.85) |
| CSF NSE (µg/L) | 3.96 (3.31- 5.31) |
| QNSE (%) | 31.4 (19.8- 40.1) |

IQR : interquartile range

**Supplementary Table S2: list of metabolites detected in the three matrices**

| **Serum** | **CSF** | **Urine** |
| --- | --- | --- |
| 1,2,3-Propanetricarboxylic | 1,2,3-Propanetricarboxylic | (2-AMINOETHYL)PHOSPHONATE |
| 12-hydroxy-9-cis-octadecenoic acid | 2-Hydroxy-4-methylpentanoic acid | (S)-1-PHENYLETHANOL |
| 17-Octadecynoic acid | 2-Hydroxyadipic acid | 1,2,3-Propanetricarboxylic |
| 2',4'-DIHYDROXYACETOPHENONE | 2-Methylbutyrylglycine | 1-Aminocyclopropanecarboxylic acid |
| 2-Hydroxy-4-methylpentanoic acid | 3-Hydroxyoctanoic acid | 1-METHYL-6,7-DIHYDROXY-1,2,3,4-TETRAHYDROISOQUINOLINE |
| 2-HYDROXYPHENYLACETIC ACID | 3-METHYL-2-OXOVALERIC ACID | 1-METHYLADENOSINE |
| 2-Methylbutyrylglycine | 4-ACETAMIDOBUTANOATE | 2',4'-DIHYDROXYACETOPHENONE |
| 3-AMINOISOBUTANOATE | 4-GUANIDINOBUTANOATE | 2,4-DIHYDROXYPTERIDINE |
| 3-hydroxydodecanoyl carnitine | 4-Hydroxyphenyllactic acid | 2,5-DIHYDROXYBENZOATE |
| 3-Hydroxyglutarate | 4-IMIDAZOLEACETIC ACID | 24-Hydroxycholesterol |
| 3-Hydroxyoctanoic acid | 5,6-DIHYDROURACIL | 2-AMINOPHENOL |
| 3-METHOXY-4-HYDROXYMANDELATE | 5-Oxoproline | 2'-DEOXYADENOSINE |
| 3-METHOXY-L-TYROSINE | 7-hydroxy-3-oxo-4-cholestenoic acid | 2'-DEOXYCYTIDINE 5'-MONOPHOSPHATE |
| 3-METHYL-2-OXOVALERIC ACID | Acetoacetic acid | 2-Hydroxyadipic acid |
| 3-Methyladipic acid | Acetyl-DL-carnitine | 2-Methylbutyrylglycine |
| 3-METHYLHISTAMINE | ADENOSINE | 3,4-DIHYDROXY-L-PHENYLALANINE |
| 4-ACETAMIDOBUTANOATE | ALPHA-AMINOADIPATE | 3,4-DIHYDROXYPHENYLACETATE |
| 4-HYDROXY-L-PROLINE | Alpha-aminobutyric acid | 3-Hydroxyadipic acid |
| 4-HYDROXYPHENYLACETATE | ALPHA-HYDROXYISOBUTYRIC ACID | 3-Hydroxyadipic acid 3,6-lactone |
| 4-Hydroxyphenyllactic acid | ANILINE | 3-HYDROXYANTHRANILATE |
| 4-METHYL-2-OXO-PENTANOIC ACID | AZELAIC ACID | 3-HYDROXYBENZOATE |
| 4-PYRIDOXATE | BETAINE | 3-hydroxyisobutyrate |
| 5-AMINOIMIDAZOLE-4-CARBOXAMIDE-1-BETA-D-RIBOFURANOSYL 5'MONOPHOSPHATE | BILIRUBIN | 3-HYDROXYKYNURENINE |
| 5-HYDROXY-L-TRYPTOPHAN | Butanoylcarnitine | 3-Hydroxyoctanoic acid |
| 5-Hydroxypipecolic acid | CADAVERINE | 3-HYDROXYPHENYLACETATE |
| 5-Methylcytosine | CAFFEINE | 3-Hydroxysuberic acid |
| 5-Oxoproline | CITRATE | 3-Methoxy-4-hydroxyhippuric acid |
| 7-hydroxy-3-oxo-4-cholestenoic acid | CITRULLINE | 3-METHOXY-4-HYDROXYMANDELATE |
| 7?-hydroxy-3-oxo-4-cholestenoic acid | CORTISOL | 3-METHOXY-L-TYROSINE |
| Acetoacetic acid | CREATINE | 3-METHYL-2-OXINDOLE |
| Acetyl-DL-carnitine | CREATININE | 3-METHYL-2-OXOVALERIC ACID |
| ADENOSINE | CYTIDINE | 3-METHYLADENINE |
| Adipic acid | D-GLUCOSAMINE | 3-Methyladipic acid |
| Adipoyl-L-carnitine | D-GLUCURONIC ACID | 3-Methylcrotonylglycine |
| Allantoin | DIHYDROXYACETONE PHOSPHATE | 3-METHYLHISTAMINE |
| Alpha-aminobutyric acid | D-PANTOTHENIC ACID | 3-Propylmalic acid |
| ALPHA-D-GLUCOSE | ETHANOLAMINE PHOSPHATE | 3-UREIDOPROPIONATE |
| ALPHA-KETOGLUTARIC ACID | GLUCONIC ACID | 4-GUANIDINOBUTANOATE |
| ANILINE | GLYCERALDEHYDE 3-PHOSPHATE DIETHYL ACETAL | 4-HYDROXY-3-METHOXYPHENYLGLYCOL |
| AZELAIC ACID | GLYCERATE | 4-HYDROXYBENZOATE |
| BENZOATE | GLYCOLATE | 4-HYDROXY-L-PROLINE |
| BENZYL ALCOHOL | HYPOXANTHINE | 4-HYDROXYPHENYLACETATE |
| BETAINE | INOSINE | 4-Hydroxyphenyllactic acid |
| BILIRUBIN | Isobutyryl-L-carnitine | 4-IMIDAZOLEACETIC ACID |
| BILIVERDIN | Isocitric acid | 4-PYRIDOXATE |
| Brassicasterol | LACTATE | 4-Pyridoxolactone |
| Butanoylcarnitine | L-ALANINE | 4-QUINOLINECARBOXYLIC ACID |
| CADAVERINE | L-ARGININE | 5,6-DIHYDROURACIL |
| CAFFEINE | L-Carnitine | 5-AMINOIMIDAZOLE-4-CARBOXAMIDE-1-BETA-D-RIBOFURANOSYL 5'-MONOPHOSPHATE |
| Cholic acid | LEUCINE | 5'-DEOXYADENOSINE |
| CITRATE | L-GLUTAMINE | 5-HYDROXYINDOLEACETATE |
| CITRULLINE | L-HISTIDINE | 5-HYDROXY-L-TRYPTOPHAN |
| CORTISOL | LL-2,6-DIAMINOHEPTANEDIOATE | 5-HYDROXYMETHYLURACIL |
| Cortisone | L-LYSINE | 5-Hydroxypipecolic acid |
| CREATINE | L-METHIONINE | 5-Methylcytosine |
| CREATININE | L-PHENYLALANINE | 5'-METHYLTHIOADENOSINE |
| CYTIDINE | L-PIPECOLIC ACID | 5-Oxoproline |
| Decanoyl-L-carnitine | L-PROLINE | 7-Dehydrocholesterol |
| Dehydrolithocholic acid | L-THREONINE | 7-Ketodeoxycholic acid |
| DEOXYCARNITINE | L-TRYPTOPHAN | ACETYLCHOLINE |
| Deoxycholic acid | L-TYROSINE | Acetyl-DL-carnitine |
| DEOXYRIBOSE | MANNITOL | ADENINE |
| D-GLUCURONIC ACID | METHYL BETA-D-GALACTOSIDE | ADENOSINE |
| DIHYDROXYACETONE PHOSPHATE | METHYL JASMONATE | Adenosine 2',3'-cyclic phosphate |
| D-LACTOSE | N(PAI)-METHYL-L-HISTIDINE | ADENOSINE 3',5'-CYCLIC MONOPHOSPHATE |
| Dodec-2-enedioic acid | N6,N6,N6-Trimethyl-L-lysine | ADENOSINE 5'-MONOPHOSPHATE |
| Dodecenoylcarnitine | N-ACETYL-D-GLUCOSAMINE | Adipic acid |
| D-PANTOTHENIC ACID | N-ACETYLGLYCINE | Adipoyl-L-carnitine |
| D-XYLOSE | N-ACETYL-L-ALANINE | Allantoin |
| Fumaric acid | N-Acetyl-L-aspartic acid | ALPHA-AMINOADIPATE |
| GLUCONIC ACID | N-ACETYLNEURAMINATE | Alpha-aminobutyric acid |
| Glutaric acid | N-Acetylserine | ALPHA-D-GLUCOSE |
| GLYCERALDEHYDE 3-PHOSPHATE DIETHYL ACETAL | N-Alpha-acetyllysine | ALPHA-KETOGLUTARIC ACID |
| GLYCERATE | PC(30_0) | ANILINE |
| GLYCEROL 2-PHOSPHATE | PC(32_0) | AZELAIC ACID |
| Glycocholic acid | PC(32_1) | BENZYL ALCOHOL |
| Glycodeoxycholic acid | PC(34_1) | BETAINE |
| GLYCOLATE | PC(34_2) | BILIVERDIN |
| Glycoursodeoxycholic acid | PC(36_1) | BIOTIN |
| Hexadecanoylcarnitine | PC(36_2) | Butanoylcarnitine |
| Hippuric acid | PC(36_3) | CADAVERINE |
| Hydroxybutyrylcarnitine | PC(38_3) | CAFFEINE |
| HYPOXANTHINE | PC(38_5) | CARNOSINE |
| INDOLE-3-ACETIC ACID | PC(38_6) | CITRULLINE |
| Indole-3-aldehyde | PC(40_6) | CORTISOL |
| Indole-3-propionic acid | Propanoylcarnitine | Cortisone |
| Indolelactic acid | Quinic acid | CREATINE |
| INDOXYL SULFATE | SARCOSINE | CREATININE |
| INOSINE | SM(34_1) | CYTIDINE |
| Isobutyryl-L-carnitine | SM(36_1) | CYTOSINE |
| Isovaleryl-L-carnitine | SM(36_2) | Decanoyl-L-carnitine |
| Kynurenic acid | SM(42_2) | DEOXYCARNITINE |
| LACTATE | SN-GLYCERO-3-PHOSPHOCHOLINE | DEOXYRIBOSE |
| L-ALANINE | SPERMIDINE | DEOXYURIDINE |
| L-ARGININE | Succinic acid | DETHIOBIOTIN |
| L-ASPARAGINE | Succinylcarnitine | D-GLUCOSAMINE |
| L-ASPARTATE | TAURINE | D-GLUCOSAMINE 6-PHOSPHATE |
| LAUROYLCARNITINE | THEOBROMINE | D-GLUCOSE 6-PHOSPHATE |
| L-Carnitine | THEOPHYLLINE | D-GLUCURONIC ACID |
| L-CYSTINE | THYMINE | DIETHANOLAMINE |
| LEUCINE | Tiglylcarnitine | D-LACTOSE |
| L-GLUTAMIC ACID | TRIGONELLINE | Dodecenoylcarnitine |
| L-GLUTAMINE | URACIL | D-PANTOTHENIC ACID |
| L-HISTIDINE | URATE | D-XYLOSE |
| Linoleyl carnitine | URIDINE | ETHANOLAMINE PHOSPHATE |
| L-KYNURENINE | Urocanic acid | FERULATE |
| L-LYSINE | Valeryl-L-carnitine | Fumaric acid |
| L-METHIONINE | XANTHINE | GALACTARATE |
| L-NORVALINE | | GLUCONIC ACID |
| L-ORNITHINE | | GLUCOSAMINATE |
| LPC(14_0) |  | Glutarylcarnitine |
| LPC(16_0) |  | GLYCERALDEHYDE 3-PHOSPHATE DIETHYL ACETAL |
| LPC(16_1) |  | GLYCERATE |
| LPC(18_0) |  | GLYCEROL 2-PHOSPHATE |
| LPC(18_2) |  | GLYCINE |
| LPC(18_4) |  | Glycocholic acid |
| LPC(20_2) |  | GLYCOLATE |
| LPC(20_3) |  | GLYOXYLIC ACID |
| LPC(20_4) |  | GUANIDINOACETATE |
| LPC(20_5) |  | GUANINE |
| LPC(22_6) |  | GUANOSINE |
| LPE(16_0) |  | GUANOSINE 3',5'-CYCLIC MONOPHOSPHATE |
| LPE(18_0) |  | Hippuric acid |
| LPE(18_2) |  | HISTAMINE |
| LPE(22_6) |  | HOMOVANILLATE |
| L-PHENYLALANINE | | HYDROQUINONE |
| L-PIPECOLIC ACID | | Hydroxybutyrylcarnitine |
| L-PROLINE |  | HYPOXANTHINE |
| LPS(22_4) |  | INDOLE-3-ACETAMIDE |
| L-SERINE |  | INDOLE-3-ACETIC ACID |
| L-THREONINE | | Indole-3-aldehyde |
| L-TRYPTOPHAN | | Indolelactic acid |
| L-TYROSINE |  | INOSINE |
| LUMICHROME | | Isocitric acid |
| L-VALINE |  | ITACONATE |
| Malic acid |  | Kynurenic acid |
| METHYL BETA-D-GALACTOSIDE | | LACTATE |
| Myristoyl-L-carnitine | | L-ALANINE |
| N(PAI)-METHYL-L-HISTIDINE | | L-ARGININE |
| N6,N6,N6-Trimethyl-L-lysine | | L-ASPARAGINE |
| N-ACETYL-DL-GLUTAMIC ACID | | L-ASPARTATE |
| N-ACETYL-D-TRYPTOPHAN | | LAUROYLCARNITINE |
| N-ACETYLGLYCINE | | L-Carnitine |
| N-ACETYL-L-ALANINE | | L-CYSTATHIONINE |
| N-Acetyl-L-aspartic acid | | L-CYSTEINE |
| N-ACETYL-L-LEUCINE | | L-CYSTINE |
| N-Acetyl-L-methionine | | L-Dihydroorotic acid |
| N-ACETYL-L-PHENYLALANINE | | LEUCINE |
| N-ACETYLNEURAMINATE | | L-GLUTAMIC ACID |
| N-ACETYLPUTRESCINE | | L-GLUTAMINE |
| N-Acetylserine | | L-HISTIDINE |
| N-Alpha-acetyllysine | | LL-2,6-DIAMINOHEPTANEDIOATE |
| NICOTINAMIDE | | L-LYSINE |
| Octadecanoylcarnitine | | L-ORNITHINE |
| Octanoyl-L-carnitine | | L-PHENYLALANINE |
| Octenoyl-L-carnitine | | L-PIPECOLIC ACID |
| Oleoylcarnitine | | L-PROLINE |
| PC(28_0) |  | L-RHAMNOSE |
| PC(30_0) |  | L-SERINE |
| PC(31_0) |  | L-THREONINE |
| PC(32_0) |  | L-TRYPTOPHAN |
| PC(32_1) |  | L-TYROSINE |
| PC(32_2) |  | LUMICHROME |
| PC(33_0) |  | L-VALINE |
| PC(33_1) |  | Maleic acid |
| PC(33_2) |  | MALEIMIDE |
| PC(34_1) |  | Malic acid |
| PC(34_3) |  | Malonyl-carnitine |
| PC(34_4) |  | MANNITOL |
| PC(35_1) |  | MESOXALATE |
| PC(35_2) |  | METHYL BETA-D-GALACTOSIDE |
| PC(35_3) |  | N(PAI)-METHYL-L-HISTIDINE |
| PC(35_4) |  | N6,N6,N6-Trimethyl-L-lysine |
| PC(36_1) |  | N-ACETYL-D-GLUCOSAMINE |
| PC(36_2) |  | N-ACETYL-DL-GLUTAMIC ACID |
| PC(36_4) |  | N-ACETYL-D-TRYPTOPHAN |
| PC(36_6) |  | N-ACETYL-L-ALANINE |
| PC(37_4) |  | N-Acetyl-L-aspartic acid |
| PC(37_5) |  | N-ACETYL-L-CYSTEINE |
| PC(37_6) |  | N-ACETYL-L-LEUCINE |
| PC(38_2) |  | N-ACETYL-L-PHENYLALANINE |
| PC(38_3) |  | N-ACETYLNEURAMINATE |
| PC(38_5) |  | N-ACETYLPUTRESCINE |
| PC(38_6) |  | N-Acetylserine |
| PC(38_7) |  | N-ACETYLSEROTONIN |
| PC(38_8) |  | N-acetyl-tyrosine |
| PC(39_6) |  | N-Alpha-acetyllysine |
| PC(40_4) |  | N-AMIDINO-L-ASPARTATE |
| PC(40_6) |  | N-FORMYLGLYCINE |
| PC(40_7) |  | NICOTINAMIDE |
| PC(40_8) |  | N-METHYLTRYPTAMINE |
| PC(42_9) |  | Octanoyl-L-carnitine |
| PC(O-14_0__2_0) | | Octenoyl-L-carnitine |
| PC(O-14_1__2_0) | | OCTOPAMINE |
| PC(O-16_3__2_0) | | Orotic acid |
| PC(O-18_3__2_0) | | PARAXANTHINE |
| PC(O-20_4__2_0) | | PHENYLACETIC ACID |
| Phenyllactic acid | | Phenyllactic acid |
| Propanoylcarnitine | | PHOSPHOCHOLINE |
| Quinic acid |  | Propanoylcarnitine |
| S-(5'-ADENOSYL)-L-HOMOCYSTEINE | | PTERIN |
| SEROTONIN |  | PYRIDOXAL |
| SM(30_1) |  | Quinic acid |
| SM(32_1) |  | RIBOFLAVIN |
| SM(32_2) |  | SARCOSINE |
| SM(33_1) |  | SM(34_1) |
| SM(34_0) |  | SN-GLYCERO-3-PHOSPHOCHOLINE |
| SM(34_1) |  | SPERMIDINE |
| SM(34_2) |  | SPERMINE |
| SM(36_1) |  | SUBERIC ACID |
| SM(36_2) |  | Succinic acid |
| SM(38_1) |  | Succinylcarnitine |
| SM(38_2) |  | TARTARIC ACID |
| SM(40_2) |  | TAURINE |
| SM(40_3) |  | Tetradecenoyl-L-carnitine |
| SM(41_2) |  | THEOBROMINE |
| SM(42_2) |  | THEOPHYLLINE |
| SM(42_3) |  | THIOPURINE S-METHYLETHER |
| SN-GLYCERO-3-PHOSPHOCHOLINE | | THYMIDINE |
| SPERMIDINE | | THYMINE |
| Succinic acid | | Tiglylcarnitine |
| Succinylcarnitine | | TRANS-CINNAMALDEHYDE |
| TAURINE |  | TRIGONELLINE |
| Taurocholic acid | | TRYPTAMINE |
| Tetradecenoyl-L-carnitine | | URACIL |
| THEOBROMINE | | URATE |
| THEOPHYLLINE | | URIDINE |
| THYROXINE |  | Uridine 5'-monophosphate |
| Tiglylcarnitine | | Urocanic acid |
| TRIGONELLINE | | Valeryl-L-carnitine |
| URACIL |  | XANTHINE |
| URATE |  | XANTHOSINE |
| URIDINE |  | Xanthurenic acid |
| Valeryl-L-carnitine | |  |
| Vanillylmandelic acid | |  |
| XANTHINE |  |  |

**Supplementary Table S3: distribution of inflammatory mediators**

| **Variable** | **Serum (pg/mL)** | **CSF (pg/mL)** |
| --- | --- | --- |
|  | **Median, IQR** | **Median, IQR** |
| b-NGF | 0.03 (0.03- 0.16) | 0.58 (0.26- 0.91) |
| CTACK | 427.66 (323.52- 540.77) | 7.31 (3.93- 9.00) |
| Eotaxin | 112.69 (83.86- 138.97) | 2.43 (2.02- 2.84) |
| FGF basic | 59.61 (41.79- 75.33) | 1.99 (1.99- 19.17) |
| G-CSF | 50.03 (29.89- 70.52) | 59.57 (47.52- 69.29) |
| GRO-a | 662.58 (591.73- 722.02) | 9.92 (9.92- 49.61) |
| HGF | 328.08 (270.76- 386.70) | 131.87 (105.77- 170.83) |
| IFN-a2 | 0.44 (0.44- 2.20) |  |
| IFN-g | 16.02 (12.48- 19.62) | 10.26 (8.35- 13.14) |
| IL-1a | 12.78 (12.78- 18.42) | 86.59 (60.32- 103.08) |
| IL-1b | 7.74 (6.20- 9.67) | 8.43 (5.80- 11.71) |
| IL-1ra | 141.01 (110.70- 195.39) | 305.68 (194.82- 428.52) |
| IL-2 | 0.00 (0.00- 0.00) | 1.27 (0.48- 2.06) |
| IL-2Ra | 31.48 (24.15- 40.98) | 3.95 (3.12- 4.79) |
| IL-3 | 0.01 (0.01- 0.01) | 0.07 (0.00- 0.16) |
| IL-4 | 0.06 (0.06- 0.28) | 0.35 (0.07- 0.83) |
| IL-5 | 0.04 (0.04- 0.66) | 103.91 (66.45- 149.56) |
| IL-6 | 26.13 (16.60- 28.34) | 3.87 (3.30- 4.96) |
| IL-7 | 6.57 (5.31- 11.04) | 0.87 (0.87- 10.49) |
| IL-8 | 496.44 (448.79- 537.10) | 57.92 (47.69- 76.02) |
| IL-9 | 5.66 (2.78- 8.45) | 15.94 (2.75- 31.40) |
| IL-10 | 0.04 (0.04- 0.66) | 29.01 (17.50- 37.18) |
| IL-12_p70 | 0.05 (0.05- 0.05) | 0.05 (0.05- 0.05) |
| IL-12_p40 |  | 1.19 (1.19- 15.79) |
| IL-13 | 0.75 (0.22- 1.19) | 3.77 (2.51- 5.14) |
| IL-15 |  | 169.21 (64.29- 226.03) |
| IL-16 | 23.37 (15.21- 33.80) | 6.98 (4.44- 9.45) |
| IL-17A | 7.97 (6.52- 9.43) | 3.00 (1.28- 4.04) |
| IL-18 | 37.37 (28.61- 52.10) | 1.47 (0.16- 3.21) |
| IP-10 | 190.12 (143.79- 256.06) | 575.02 (441.27- 800.86) |
| LIF | 68.03 (3.44- 92.62) | 140.56 (56.95- 202.65) |
| M-CSF | 6.86 (5.21- 8.95) | 21.52 (16.81- 25.08) |
| MCP-1(MCAF) | 16.85 (11.79- 23.06) | 161.74 (136.70- 202.66) |
| MCP-3 | 0.10 (0.10- 0.48) | 8.74 (5.51- 12.10) |
| MIF | 501.02 (354.86- 655.78) | 424.14 (341.44- 505.04) |
| MIG | 118.48 (78.67- 185.06) | 9.20 (9.20- 9.20) |
| MIP-1a | 1.25 (0.90- 1.95) | 0.04 (0.04- 0.43) |
| MIP-1b | 172.58 (159.14- 187.80) | 5.81 (4.75- 6.71) |
| PDGF-bb | 1002.00 (683.12- 1284.39) | 54.91 (35.01- 73.07) |
| RANTES | 7195.18 (5912.73- 8585.96) | 12.11 (9.85- 14.32) |
| SCF | 108.43 (94.54- 127.10) | 36.62 (30.00- 42.46) |
| SCGF-b | 117425.67 (104081.52- 134306.86) | 36688.71 (27785.44- 46913.77) |
| SDF-1a | 817.21 (732.10- 893.90) | 774.20 (594.23- 1024.38) |
| TNF-a | 57.12 (51.53- 68.17) | 59.51 (42.81- 78.49) |
| TNF-b | 335.14 (314.48- 364.46) | 36.14 (26.70- 43.83) |
| TRAIL | 32.06 (28.52- 35.83) | 2.18 (1.68- 3.75) |
| VEGF |  | 6.31 (2.10- 77.20) |

IQR : interquartile range

**Supplementary Table S3B: distribution of Kynurenine pathway metabolites**

| **Variable** | **Serum** | **CSF** |
| --- | --- | --- |
|  | **Median, IQR** | **Median, IQR** |
| Picolinic acid (nmol/L) | 7.78 (5.49- 11.06) | 5.37 (0.76- 8.20) |
| Quinolinic acid (nmol/L) | 202.37 (155.96- 240.64) | 2.51 (2.51- 17.99) |
| 3-OH-Kynurenine (nmol/L) | 28.22 (20.36- 37.97) | 2.05 (2.05- 11.11) |
| Serotonine (nmol/L) | 180.46 (138.25- 228.17) |  |
| 5-OH-Tryptophane (nmol/L) | 5.25 (4.73- 5.63) | 8.21 (6.90- 9.17) |
| Kynurenine (nmol/L) | 1433.11 (1212.87- 1710.30) | 21.89 (11.63- 38.86) |
| 3-OH-Anthranilic acid (nmol/L) | 57.01 (46.80- 69.42) | 21.39 (18.15- 25.26) |
| Tryptamine (nmol/L) | 1.21 (0.97- 1.61) | 2130.33 (1833.88- 2458.03) |
| Tryptophane (nmol/L) | 53542.05 (47547.90- 59077.88) | 92.94 (60.98- 121.10) |
| 5-OH-Indole acetic acid (nmol/L) | 25.46 (21.51- 34.31) |  |
| Indole-3-Sulfate (nmol/L) | 2681.07 (1956.68- 3418.74) |  |
| N-acetyl-serotonine (nmol/L) | 0.03 (0.03- 0.03) |  |
| Xanthurenic acid (nmol/L) | 7.14 (5.45- 9.55) |  |
| Indole-3-acetamide (nmol/L) | 0.10 (0.10- 0.10) | 0.24 (0.24- 0.24) |
| Kynurenic Acid (nmol/L) | 14.36 (11.02- 18.70) | 0.18 (0.18- 0.18) |
| Indole-3-Aldehyde (nmol/L) | 1.13 (1.13- 7.07) |  |
| Indole-3-Lactic acid (nmol/L) | 815.58 (616.41- 950.96) |  |
| Indole-3-Acetic acid (nmol/L) | 990.76 (804.22- 1228.83) | 13.08 (9.17- 17.26) |
| KYN_TRP | 0.03 (0.02- 0.03) | 0.01 (0.01- 0.02) |
| KYNA_KYN | 0.01 (0.01- 0.01) | 0.01 (0.01- 0.02) |
| 3-HK_KYN | 0.02 (0.02- 0.02) | 0.15 (0.06- 0.81) |
| QUIN_KYNA | 13.66 (10.74- 16.56) | 14.03 (14.03- 71.03) |
| QUIN_KYN | 0.14 (0.12- 0.16) | 0.27 (0.12- 0.84) |
| 3-HK_KYNA | 1.92 (1.53- 2.46) | 11.47 (11.47- 59.67) |

IQR : interquartile range

**Supplementary Table S4: Features associated with age of onset**

| **Overall** |  |  | **Male** |  |  | **Female** |  |  |
| --- | --- | --- | --- | --- | --- | --- | --- | --- |
| **Feature** | **r** | **FDR** | **Feature** | **r** | **FDR** | **Feature** | **r** | **FDR** |
| MIG_Serum | 0.52 | <0.001 | MIG_CSF | 0.61 | <0.001 | GRO-a_Serum | -0.68 | 0.032 |
| MIG_CSF | 0.50 | <0.001 | SCF_CSF | 0.59 | <0.001 |  |  |  |
| MIP-1b_CSF | 0.40 | 0.015 | MIG_Serum | 0.56 | 0.002 |  |  |  |
| SCF_CSF | 0.39 | 0.015 | IFN-g_CSF | 0.50 | 0.005 |  |  |  |
| TNF-a_CSF | 0.39 | 0.015 | IL-1a_CSF | 0.51 | 0.005 |  |  |  |
| MIP-1b_Ratio | 0.41 | 0.015 | IL-17A_CSF | 0.50 | 0.005 |  |  |  |
| M-CSF_Serum | 0.35 | 0.048 | M-CSF_CSF | 0.50 | 0.005 |  |  |  |
| IL-8_CSF | 0.34 | 0.048 | MIF_CSF | 0.52 | 0.005 |  |  |  |
| M-CSF_CSF | 0.34 | 0.048 | MIP-1b_CSF | 0.51 | 0.005 |  |  |  |
| N6,N6,N6-Trimethyl-L-lysine_CSF | -0.66 | <0.001 | LIF_CSF | 0.46 | 0.013 |  |  |  |
| Myristoyl-L-carnitine_Serum | 0.53 | <0.001 | TNF-a_CSF | 0.46 | 0.013 |  |  |  |
| INOSINE_CSF | -0.53 | <0.001 | MCP-3_CSF | 0.45 | 0.016 |  |  |  |
| SN-GLYCERO-3-PHOSPHOCHOLINE_CSF | -0.53 | <0.001 | MIP-1b_Ratio | 0.45 | 0.016 |  |  |  |
| Tiglylcarnitine_CSF | 0.53 | <0.001 | CTACK_CSF | 0.44 | 0.018 |  |  |  |
| Hexadecanoylcarnitine_Serum | 0.51 | 0.002 | IL-1b_CSF | 0.44 | 0.019 |  |  |  |
| Oleoylcarnitine_Serum | 0.49 | 0.003 | IL-17A_Ratio | 0.42 | 0.030 |  |  |  |
| Tetradecenoyl-L-carnitine_Serum | 0.45 | 0.016 | M-CSF_Serum | 0.40 | 0.036 |  |  |  |
| L-METHIONINE_Serum | -0.43 | 0.028 | TRAIL_CSF | 0.40 | 0.036 |  |  |  |
| Hydroxybutyrylcarnitine_Serum | 0.42 | 0.034 | FGF basic_CSF | 0.40 | 0.037 |  |  |  |
| Quinolinic acid_CSF | 0.48 | <0.001 | IL-13_CSF | 0.40 | 0.037 |  |  |  |
| Indole-3-Acetic acid_Ratio | 0.48 | <0.001 | G-CSF_Serum | 0.39 | 0.038 |  |  |  |
| Picolinic acid_Ratio | 0.47 | <0.001 | IL-16_CSF | 0.39 | 0.038 |  |  |  |
| Kynurenine_Ratio | 0.45 | <0.001 | IL-10_CSF | 0.39 | 0.038 |  |  |  |
| Kynurenine_CSF | 0.44 | 0.001 | b-NGF_CSF | 0.38 | 0.043 |  |  |  |
| KYN_TRP_CSF | 0.42 | 0.002 | TRAIL_Ratio | 0.38 | 0.043 |  |  |  |
| Kynurenic Acid_CSF | 0.38 | 0.007 | SCGF-b_Ratio | 0.38 | 0.043 |  |  |  |
| Indole-3-Acetic acid_CSF | 0.38 | 0.007 | TNF-b_CSF | 0.38 | 0.044 |  |  |  |
| Picolinic acid_CSF | 0.37 | 0.008 | IL-4_CSF | 0.37 | 0.045 |  |  |  |
| Quinolinic acid_Ratio | 0.37 | 0.008 | IL-1ra_CSF | 0.37 | 0.046 |  |  |  |
| 3-HK_KYN_CSF | -0.35 | 0.014 | N6,N6,N6-Trimethyl-L-lysine_CSF | -0.72 | <0.001 |  |  |  |
| Tryptophane_Ratio | 0.34 | 0.018 | INOSINE_CSF | -0.58 | 0.008 |  |  |  |
| QUIN_KYN_Serum | 0.32 | 0.026 | SN-GLYCERO-3-PHOSPHOCHOLINE_CSF | -0.59 | 0.008 |  |  |  |
| Quinolinic acid_Serum | 0.32 | 0.027 | Tiglylcarnitine_CSF | 0.54 | 0.027 |  |  |  |
|  |  |  | Oleoylcarnitine_Serum | 0.52 | 0.040 |  |  |  |
|  |  |  | Hydroxybutyrylcarnitine_Serum | 0.51 | 0.041 |  |  |  |
|  |  |  | Indole-3-Acetic acid_Ratio | 0.65 | <0.001 |  |  |  |
|  |  |  | Kynurenine_Ratio | 0.57 | <0.001 |  |  |  |
|  |  |  | Kynurenine_CSF | 0.56 | <0.001 |  |  |  |
|  |  |  | Picolinic acid_Ratio | 0.55 | <0.001 |  |  |  |
|  |  |  | Picolinic acid_CSF | 0.52 | 0.002 |  |  |  |
|  |  |  | KYN_TRP_CSF | 0.52 | 0.002 |  |  |  |
|  |  |  | Indole-3-Acetic acid_CSF | 0.51 | 0.002 |  |  |  |
|  |  |  | Quinolinic acid_CSF | 0.44 | 0.013 |  |  |  |
|  |  |  | Kynurenic Acid_CSF | 0.43 | 0.013 |  |  |  |
|  |  |  | Tryptophane_Ratio | 0.40 | 0.030 |  |  |  |

**Supplementary Table S5: Features associated with ALSFRS-R at diagnostic**

| **Overall** |  |  | **Male** |  |  | **Female** |  |  |
| --- | --- | --- | --- | --- | --- | --- | --- | --- |
| **Feature** | **r** | **FDR** | **Feature** | **r** | **FDR** | **Feature** | **r** | **FDR** |
| NfL_Serum | -0.40 | 0.001 | S100_CSF | -0.46 | 0.01 | NfL_Serum | -0.57 | 0.006 |
| NfL_CSF | -0.37 | 0.001 | SM(36_2)_Serum | -0.62 | 0.004 | NfL_CSF | -0.57 | 0.006 |
| S100_CSF | -0.44 | 0.002 | SN-GLYCERO-3-PHOSPHOCHOLINE_LCR | 0.55 | 0.042 | IL-2_Ratio | 0.70 | 0.029 |
|  |  |  | SM(34_2)_Serum | -0.53 | 0.047 | N-ACETYL-D-TRYPTOPHAN_Serum | -0.86 | <0.001 |
|  |  |  | SM(38_2)_Serum | -0.52 | 0.047 |  |  |  |

**Supplementary Table S6: Features associated with weight at diagnostic**

| **Overall** |  |  | **Male** |  |  | **Female** |  |  |
| --- | --- | --- | --- | --- | --- | --- | --- | --- |
| **Feature** | **r** | **FDR** | **Feature** | **r** | **FDR** | **Feature** | **r** | **FDR** |
| NfL_Serum | -0.38 | 0.002 | NfL_Serum | -0.38 | 0.018 |  |  |  |
| Valeryl-L-carnitine_Serum | 0,55 | 0,001 | NfL_CSF | -0.33 | 0 .025 |  |  |  |
| PC(35_2)_Serum | -0,52 | 0,003 |  |  |  |  |  |  |
| PC(33_2)_Serum | -0,48 | 0,014 |  |  |  |  |  |  |
| 5-AMINOIMIDAZOLE-4-CARBOXAMIDE-1-BETA-D-RIBOFURANOSYL 5'-MONOPHOSPHATE_Serum | 0,46 | 0,015 |  |  |  |  |  |  |
| Isovaleryl-L-carnitine_Serum | 0,46 | 0,015 |  |  |  |  |  |  |
| PC(35_1)_Serum | -0,43 | 0,033 |  |  |  |  |  |  |
| L-TYROSINE_Serum | 0,40 | 0,048 |  |  |  |  |  |  |
| N-ACETYLGLYCINE_Serum | -0,40 | 0,048 |  |  |  |  |  |  |
| 2-Hydroxyadipic acid_Urine | 0,41 | 0,048 |  |  |  |  |  |  |
| 3,4-DIHYDROXYPHENYLACETATE_Urine | 0,40 | 0,048 |  |  |  |  |  |  |
| 5-Oxoproline_Urine | -0,41 | 0,048 |  |  |  |  |  |  |
| GUANIDINOACETATE_Urine | -0,40 | 0,048 |  |  |  |  |  |  |
| SPERMIDINE_Urine | 0,42 | 0,048 |  |  |  |  |  |  |
| Xanthurenic acid_Serum | 0.41 | 0.017 |  |  |  |  |  |  |
| Tryptophane_Serum | 0.39 | 0.018 |  |  |  |  |  |  |
| Kynurenic Acid_Serum | 0.35 | 0.045 |  |  |  |  |  |  |
| Kynurenic Acid_Ratio | -0.34 | 0.049 |  |  |  |  |  |  |

**Supplementary Table S7: Features associated with variation of reference weight at diagnostic**

| **Overall** |  |  | **Male** |  |  | **Female** |  |  |
| --- | --- | --- | --- | --- | --- | --- | --- | --- |
| **Feature** | **r** | **FDR** | **Feature** | **r** | **FDR** | **Feature** | **r** | **FDR** |
|  |  |  | Dodec-2-enedioic acid_Serum | -0.66 | 0.009 |  |  |  |
|  |  |  | Urocanic acid_Urine | 0.62 | 0.023 |  |  |  |

**Supplementary Table S8: Features associated with FVC at diagnostic**

| **Overall** |  |  | **Male** |  |  | **Female** |  |  |
| --- | --- | --- | --- | --- | --- | --- | --- | --- |
| **Feature** | **r** | **FDR** | **Feature** | **r** | **FDR** | **Feature** | **r** | **FDR** |
| S100_serum | -0.37 | 0.003 |  |  |  | S100_serum | -0.59 | 0.021 |
| QS100 | 0.34 | 0.006 |  |  |  |  |  |  |
| PC(32_1)_Ratio | -0.59 | <0.001 |  |  |  |  |  |  |
| PC(32_1)_Serum | 0.55 | 0.003 |  |  |  |  |  |  |
| PC(36_6)_Serum | 0.53 | 0.005 |  |  |  |  |  |  |

**Supplementary Table S9: Features associated with variation of ALSFRS-R over a year**

| **Overall** |  |  | **Male** |  |  | **Female** |  |  |
| --- | --- | --- | --- | --- | --- | --- | --- | --- |
| **Feature** | **r** | **FDR** | **Feature** | **r** | **FDR** | **Feature** | **r** | **FDR** |
| NfL_Serum | -0.5 | 0.002 | NfL_Serum | -0.59 | 0.001 |  |  |  |
| NfL_CSF | -0.49 | 0.003 | NfL_CSF | -0.45 | 0.013 |  |  |  |

**Supplementary Table S10: Features associated with variation of FVC over a year**

| **Overall** |  |  | **Male** |  |  | **Female** |  |  |
| --- | --- | --- | --- | --- | --- | --- | --- | --- |
| **Feature** | **r** | **FDR** | **Feature** | **r** | **FDR** | **Feature** | **r** | **FDR** |
| NfL_Serum | -0.61 | 0.011 |  |  |  | NfL_Serum | -0.83 | 0.001 |
| NfL_CSF | -0.42 | 0.041 |  |  |  | NfL_CSF | -0.83 | 0.013 |
|  |  |  |  |  |  | Albumin_serum | 0.94 | 0.043 |
|  |  |  |  |  |  | 7’-hydroxy-3-oxo-4-cholestenoic acid_Serum | 1 | <0.001 |
|  |  |  |  |  |  | PC(40:6)_Serum | 1 | <0.001 |
|  |  |  |  |  |  | N-ACETYL-L-ALANINE_CSF | 1 | <0.001 |
|  |  |  |  |  |  | 3-UREIDOPROPIONATE_Urine | 1 | <0.001 |
|  |  |  |  |  |  | 5-Oxoproline_Urine | 1 | <0.001 |

**Supplementary Table S11: VIP of features included in the PLS model to explain serum NfL levels**

| **Feature** | **VIP** |
| --- | --- |
| D-PANTOTHENIC ACID_Ratio | 1.41 |
| S100_LCR | 1.40 |
| N-METHYLTRYPTAMINE_Urine | 1.34 |
| Valeryl-L-carnitine_Ratio | 1.32 |
| 5-OH-Indole acetic acid_Serum | 1.29 |
| THYMIDINE_Urine | 1.29 |
| N-ACETYL-D-TRYPTOPHAN_Serum | 1.29 |
| PC(37_4)_Serum | 1.24 |
| PC(39_6)_Serum | 1.24 |
| L-CYSTINE_Serum | 1.23 |
| GLUCOSAMINATE_Urine | 1.21 |
| GUANOSINE 3',5'-CYCLIC MONOPHOSPHATE_Urine | 1.17 |
| 5-OH-Tryptophane_Serum | 1.17 |
| INDOLE-3-ACETIC ACID_Serum | 1.15 |
| PC(35_2)_Serum | 1.14 |
| D-PANTOTHENIC ACID_LCR | 1.12 |
| Isocitric acid_LCR | 1.12 |
| Glycodeoxycholic acid_Serum | 1.10 |
| Valeryl-L-carnitine_LCR | 1.10 |
| 4-IMIDAZOLEACETIC ACID_LCR | 1.10 |
| TAURINE_LCR | 1.07 |
| Indole-3-Acetic acid_Serum | 1.06 |
| Tiglylcarnitine_Ratio | 1.05 |
| 3-METHYLADENINE_Urine | 1.05 |
| N-Acetylserine_Urine | 1.03 |
| Butanoylcarnitine_Ratio | 1.03 |
| 5-Oxoproline_LCR | 1.00 |
| Indolelactic acid_Serum | 1.00 |
| Maleic acid_Urine | 0.99 |
| IL-18_Serum | 0.99 |
| Isobutyryl-L-carnitine_LCR | 0.99 |
| LEUCINE_LCR | 0.98 |
| 17-Octadecynoic acid_Serum | 0.97 |
| PC(37_5)_Serum | 0.96 |
| PC(40_7)_Serum | 0.95 |
| Dodec-2-enedioic acid_Serum | 0.95 |
| MCP-1(MCAF)_LCR | 0.95 |
| Indole-3-Aldehyde_Serum | 0.95 |
| L-TRYPTOPHAN_Serum | 0.95 |
| 5-HYDROXYMETHYLURACIL_Urine | 0.95 |
| 3-METHOXY-L-TYROSINE_Serum | 0.94 |
| Propanoylcarnitine_Ratio | 0.94 |
| IL-8_Ratio | 0.93 |
| L-CYSTINE_Urine | 0.93 |
| TAURINE_Ratio | 0.93 |
| LPE(18_0)_Serum | 0.92 |
| 3-OH-Anthranilic acid_LCR | 0.91 |
| PC(35_4)_Serum | 0.90 |
| 1,2,3-Propanetricarboxylic_LCR | 0.90 |
| IL-3_LCR | 0.89 |
| SM(30_1)_Serum | 0.89 |
| Glutaric acid_Serum | 0.89 |
| b-NGF_LCR | 0.89 |
| GLYCERATE_Serum | 0.88 |
| 3-METHYL-2-OXOVALERIC ACID_LCR | 0.88 |
| HYPOXANTHINE_LCR | 0.88 |
| 3-OH-Anthranilic acid_Ratio | 0.88 |
| L-VALINE_Serum | 0.87 |
| L-Dihydroorotic acid_Urine | 0.87 |
| SM(42_2)_Ratio | 0.87 |
| Dodecenoylcarnitine_Urine | 0.87 |
| PC(38_5)_Serum | 0.86 |
| IL-3_Ratio | 0.86 |
| ITACONATE_Urine | 0.85 |
| L-ASPARAGINE_Serum | 0.85 |
| Butanoylcarnitine_LCR | 0.85 |
| SUBERIC ACID_Urine | 0.85 |
| 5-AMINOIMIDAZOLE-4-CARBOXAMIDE-1-BETA-D-RIBOFURANOSYL 5'-MONOPHOSPHATE_Urine | 0.85 |
| 2,5-DIHYDROXYBENZOATE_Urine | 0.85 |
| GLYCERATE_LCR | 0.84 |
| L-TRYPTOPHAN_Urine | 0.84 |
| 3-HYDROXYKYNURENINE_Urine | 0.83 |
| Picolinic acid_Ratio | 0.83 |
| L-TYROSINE_Urine | 0.83 |
| b-NGF_Ratio | 0.81 |
| 5-OH-Indole acetic acid_Ratio | 0.81 |
| Adipic acid_Serum | 0.81 |
| 5-Oxoproline_Ratio | 0.81 |
| MCP-1(MCAF)_Ratio | 0.80 |
| PC(37_6)_Serum | 0.80 |

**Supplementary Table S12: Features associated with QAlb**

| **Overall** |  |  |  |
| --- | --- | --- | --- |
| **Feature** | **Block** | **r** | **FDR** |
| MIP-1a_CSF | Inflammation | 0.50 | 0.001 |
| IP-10_CSF | Inflammation | 0.46 | 0.003 |
| SCGF-b_CSF | Inflammation | 0.44 | 0.003 |
| TNF-b_CSF | Inflammation | 0.44 | 0.003 |
| TNF-b_Ratio | Inflammation | 0.45 | 0.003 |
| IL-8_CSF | Inflammation | 0.42 | 0.004 |
| IL-10_CSF | Inflammation | 0.38 | 0.017 |
| IL-1b_Ratio | Inflammation | 0.38 | 0.017 |
| HGF_CSF | Inflammation | 0.37 | 0.018 |
| IL-16_CSF | Inflammation | 0.36 | 0.018 |
| M-CSF_CSF | Inflammation | 0.37 | 0.018 |
| MCP-3_CSF | Inflammation | 0.36 | 0.018 |
| MIP-1a_Ratio | Inflammation | 0.36 | 0.018 |
| IP-10_Ratio | Inflammation | 0.34 | 0.034 |
| IL-1b_CSF | Inflammation | 0.34 | 0.035 |
| SM(42_2)_CSF | Metabolome | 0.81 | <0.001 |
| SM(42_2)_Ratio | Metabolome | 0.78 | <0.001 |
| Acetyl-DL-carnitine _Ratio | Metabolome | 0.77 | <0.001 |
| Acetyl-DL-carnitine _CSF | Metabolome | 0.75 | <0.001 |
| 7-hydroxy-3-oxo-4-cholestenoic acid_Ratio | Metabolome | 0.74 | <0.001 |
| PC(34_2)_CSF | Metabolome | 0.74 | <0.001 |
| PC(36_2)_CSF | Metabolome | 0.74 | <0.001 |
| PC(36_2)_Ratio | Metabolome | 0.74 | <0.001 |
| SM(34_1)_Ratio | Metabolome | 0.72 | <0.001 |
| PC(36_3)_CSF | Metabolome | 0.72 | <0.001 |
| PC(38_5)_Ratio | Metabolome | 0.72 | <0.001 |
| SM(34_1)_CSF | Metabolome | 0.71 | <0.001 |
| PC(38_3)_Ratio | Metabolome | 0.71 | <0.001 |
| PC(38_3)_CSF | Metabolome | 0.71 | <0.001 |
| PC(38_5)_CSF | Metabolome | 0.70 | <0.001 |
| Propanoylcarnitine_CSF | Metabolome | 0.69 | <0.001 |
| 7-hydroxy-3-oxo-4-cholestenoic acid_CSF | Metabolome | 0.67 | <0.001 |
| HYPOXANTHINE_CSF | Metabolome | -0.67 | <0.001 |
| Propanoylcarnitine_Ratio | Metabolome | 0.66 | <0.001 |
| L-Carnitine_CSF | Metabolome | 0.65 | <0.001 |
| URATE_CSF | Metabolome | 0.64 | <0.001 |
| SM(36_2)_CSF | Metabolome | 0.62 | <0.001 |
| L-PROLINE_Ratio | Metabolome | 0.61 | <0.001 |
| PC(38_6)_Ratio | Metabolome | 0.61 | <0.001 |
| PC(38_6)_CSF | Metabolome | 0.61 | <0.001 |
| L-Carnitine_Ratio | Metabolome | 0.61 | <0.001 |
| L-PROLINE_CSF | Metabolome | 0.59 | <0.001 |
| URATE_Ratio | Metabolome | 0.59 | <0.001 |
| PC(30_0)_CSF | Metabolome | 0.59 | <0.001 |
| PC(40_6)_CSF | Metabolome | 0.58 | <0.001 |
| 4-ACETAMIDOBUTANOATE_CSF | Metabolome | -0.58 | <0.001 |
| ETHANOLAMINE PHOSPHATE_CSF | Metabolome | 0.57 | <0.001 |
| XANTHINE_CSF | Metabolome | -0.57 | <0.001 |
| SM(36_2)_Ratio | Metabolome | 0.56 | <0.001 |
| N-Acetyl-L-aspartic acid_CSF | Metabolome | -0.56 | <0.001 |
| L-TYROSINE_Ratio | Metabolome | 0.56 | <0.001 |
| PC(40_6)_Ratio | Metabolome | 0.55 | <0.001 |
| PC(36_1)_CSF | Metabolome | 0.54 | <0.001 |
| L-ALANINE_Ratio | Metabolome | 0.54 | <0.001 |
| PC(32_1)_CSF | Metabolome | 0.54 | <0.001 |
| PC(36_1)_Ratio | Metabolome | 0.54 | <0.001 |
| L-ALANINE_CSF | Metabolome | 0.53 | <0.001 |
| BILIRUBIN_Ratio | Metabolome | 0.53 | <0.001 |
| Butanoylcarnitine_CSF | Metabolome | 0.53 | <0.001 |
| CREATININE_CSF | Metabolome | -0.53 | <0.001 |
| N-Acetyl-L-aspartic acid_Ratio | Metabolome | -0.52 | <0.001 |
| SM(36_1)_CSF | Metabolome | 0.50 | <0.001 |
| Valeryl-L-carnitine_CSF | Metabolome | 0.49 | <0.001 |
| BILIRUBIN_CSF | Metabolome | 0.48 | 0.001 |
| N-ACETYL-L-ALANINE_CSF | Metabolome | -0.48 | 0.001 |
| PC(30_0)_Ratio | Metabolome | 0.47 | 0.001 |
| HYPOXANTHINE_Ratio | Metabolome | -0.47 | 0.001 |
| N-Alpha-acetyllysine_Ratio | Metabolome | 0.46 | 0.002 |
| PC(34_1)_CSF | Metabolome | 0.46 | 0.002 |
| CITRATE_CSF | Metabolome | 0.45 | 0.002 |
| N-ACETYL-L-ALANINE_Ratio | Metabolome | -0.44 | 0.003 |
| L-TYROSINE_CSF | Metabolome | 0.44 | 0.003 |
| L-HISTIDINE_Ratio | Metabolome | 0.43 | 0.004 |
| 2-Methylbutyrylglycine_CSF | Metabolome | -0.43 | 0.004 |
| L-PHENYLALANINE_CSF | Metabolome | 0.42 | 0.005 |
| 4-ACETAMIDOBUTANOATE_Ratio | Metabolome | -0.41 | 0.007 |
| L-PHENYLALANINE_Ratio | Metabolome | 0.41 | 0.007 |
| AZELAIC ACID_CSF | Metabolome | -0.41 | 0.007 |
| L-THREONINE_Ratio | Metabolome | 0.41 | 0.007 |
| SM(36_1)_Ratio | Metabolome | 0.40 | 0.011 |
| N-Acetylserine_CSF | Metabolome | -0.39 | 0.012 |
| Butanoylcarnitine_Ratio | Metabolome | 0.39 | 0.013 |
| PC(32_0)_CSF | Metabolome | 0.39 | 0.015 |
| LEUCINE_CSF | Metabolome | 0.38 | 0.019 |
| XANTHINE_Ratio | Metabolome | -0.37 | 0.021 |
| Valeryl-L-carnitine_Ratio | Metabolome | 0.37 | 0.022 |
| CYTIDINE_CSF | Metabolome | -0.37 | 0.022 |
| PC(34_1)_Ratio | Metabolome | 0.37 | 0.022 |
| 2-Hydroxyadipic acid_CSF | Metabolome | -0.37 | 0.024 |
| 3-Hydroxyoctanoic acid_CSF | Metabolome | -0.36 | 0.026 |
| N(PAI)-METHYL-L-HISTIDINE_Ratio | Metabolome | 0.36 | 0.026 |
| L-METHIONINE_Ratio | Metabolome | 0.36 | 0.029 |
| BETAINE_Ratio | Metabolome | 0.36 | 0.030 |
| Alpha-aminobutyric acid_CSF | Metabolome | 0.36 | 0.031 |
| D-PANTOTHENIC ACID_Ratio | Metabolome | -0.35 | 0.034 |
| CITRATE_Ratio | Metabolome | 0.35 | 0.035 |
| N(PAI)-METHYL-L-HISTIDINE_CSF | Metabolome | 0.35 | 0.038 |
| 4-Hydroxyphenyllactic acid_CSF | Metabolome | -0.34 | 0.047 |
| METHYL JASMONATE_CSF | Metabolome | -0.34 | 0.047 |
| LEUCINE_Ratio | Metabolome | 0.34 | 0.048 |

**Supplementary Table S13: VIP of features included in the PLS model to explain QAlb levels**

| **Feature** | **VIP** |
| --- | --- |
| PC(36_2)_CSF | 1.19 |
| PC(36_2)_Ratio | 1.18 |
| SM(34_1)_CSF | 1.17 |
| SM(34_1)_Ratio | 1.16 |
| PC(34_2)_CSF | 1.14 |
| SM(42_2)_CSF | 1.14 |
| PC(38_3)_Ratio | 1.13 |
| SM(42_2)_Ratio | 1.12 |
| PC(38_3)_CSF | 1.11 |
| 7-hydroxy-3-oxo-4-cholestenoic acid_CSF | 1.08 |
| PC(38_5)_Ratio | 1.07 |
| 7-hydroxy-3-oxo-4-cholestenoic acid_Ratio | 1.06 |
| PC(38_5)_CSF | 1.05 |
| PC(36_3)_CSF | 1.05 |
| PC(38_6)_CSF | 1.03 |
| PC(38_6)_Ratio | 1.00 |
| PC(32_1)_CSF | 0.96 |
| SM(36_2)_CSF | 0.94 |
| PC(36_1)_CSF | 0.93 |
| PC(40_6)_CSF | 0.91 |
| SM(36_2)_Ratio | 0.90 |
| SM(36_1)_CSF | 0.87 |
| Acetyl-DL-carnitine _CSF | 0.87 |
| Acetyl-DL-carnitine _Ratio | 0.87 |
| PC(40_6)_Ratio | 0.85 |
| PC(34_1)_CSF | 0.84 |
| PC(36_1)_Ratio | 0.84 |
| Propanoylcarnitine_Ratio | 0.83 |
| Propanoylcarnitine_CSF | 0.83 |
| PC(30_0)_CSF | 0.83 |
| CITRATE_CSF | 0.81 |

**Supplementary Table S14: Features associated with CSF NSE**

| **Overall** |  |  |  |
| --- | --- | --- | --- |
| **Feature** | **Block** | **r** | **FDR** |
| MIF_CSF | Inflammation | 0.79 | <0.001 |
| SCF_CSF | Inflammation | 0.56 | <0.001 |
| M-CSF_CSF | Inflammation | 0.53 | <0.001 |
| MIF_Ratio | Inflammation | 0.52 | <0.001 |
| IFN-g_Ratio | Inflammation | 0.50 | <0.001 |
| IFN-g_CSF | Inflammation | 0.47 | 0.001 |
| TNF-b_CSF | Inflammation | 0.47 | 0.001 |
| TNF-b_Ratio | Inflammation | 0.48 | 0.001 |
| G-CSF_CSF | Inflammation | 0.44 | 0.002 |
| SCF_Ratio | Inflammation | 0.44 | 0.002 |
| IL-16_CSF | Inflammation | 0.42 | 0.002 |
| RANTES_CSF | Inflammation | 0.42 | 0.002 |
| IL-16_Ratio | Inflammation | 0.41 | 0.004 |
| RANTES_Ratio | Inflammation | 0.41 | 0.004 |
| IL-9_CSF | Inflammation | 0.39 | 0.005 |
| LIF_CSF | Inflammation | 0.39 | 0.005 |
| IL-9_Ratio | Inflammation | 0.39 | 0.005 |
| CTACK_Ratio | Inflammation | 0.39 | 0.005 |
| MIP-1b_Ratio | Inflammation | 0.39 | 0.005 |
| IL-4_CSF | Inflammation | 0.38 | 0.006 |
| MCP-3_CSF | Inflammation | 0.36 | 0.010 |
| TNF-a_CSF | Inflammation | 0.36 | 0.010 |
| IL-18_CSF | Inflammation | 0.36 | 0.012 |
| IL-2Ra_CSF | Inflammation | 0.35 | 0.012 |
| IL-13_CSF | Inflammation | 0.35 | 0.012 |
| IL-18_Ratio | Inflammation | 0.35 | 0.013 |
| IL-2Ra_Ratio | Inflammation | 0.35 | 0.014 |
| IL-8_CSF | Inflammation | 0.34 | 0.015 |
| IL-10_CSF | Inflammation | 0.34 | 0.015 |
| IL-1b_CSF | Inflammation | 0.34 | 0.016 |
| HGF_Ratio | Inflammation | 0.34 | 0.016 |
| M-CSF_Ratio | Inflammation | 0.34 | 0.016 |
| PDGF-bb_CSF | Inflammation | 0.34 | 0.016 |
| TNF-a_Ratio | Inflammation | 0.33 | 0.016 |
| IL-17A_Ratio | Inflammation | 0.32 | 0.021 |
| MIP-1a_CSF | Inflammation | 0.32 | 0.022 |
| MIP-1b_CSF | Inflammation | 0.32 | 0.022 |
| MIP-1a_Ratio | Inflammation | 0.32 | 0.022 |
| IL-7_CSF | Inflammation | 0.31 | 0.026 |
| IL-17A_CSF | Inflammation | 0.31 | 0.030 |
| HGF_CSF | Inflammation | 0.30 | 0.030 |
| Eotaxin_Ratio | Inflammation | 0.30 | 0.030 |
| IL-4_Ratio | Inflammation | 0.30 | 0.031 |
| LIF_Ratio | Inflammation | 0.29 | 0.037 |
| IL-3_Ratio | Inflammation | 0.29 | 0.037 |
| CTACK_CSF | Inflammation | 0.29 | 0.042 |
| PDGF-bb_Ratio | Inflammation | 0.28 | 0.046 |
| IL-3_CSF | Inflammation | 0.28 | 0.046 |
| IL-12_p70_Ratio | Inflammation | 0.28 | 0.047 |
| IFN-g_Serum | Inflammation | -0.28 | 0.049 |
| IP-10_Ratio | Inflammation | 0.28 | 0.049 |
| N-ACETYLNEURAMINATE_CSF | Metabolome | 0.51 | 0.006 |
| N-Acetylserine_CSF | Metabolome | 0.51 | 0.006 |
| CAFFEINE_Urine | Metabolome | 0.50 | 0.006 |
| 2-Methylbutyrylglycine_CSF | Metabolome | 0.49 | 0.007 |
| N-Acetylserine_Ratio | Metabolome | 0.47 | 0.008 |
| 2'-DEOXYCYTIDINE 5'-MONOPHOSPHATE_Urine | Metabolome | 0.47 | 0.008 |
| PC(30_0)_Serum | Metabolome | 0.45 | 0.015 |
| L-CYSTINE_Serum | Metabolome | 0.44 | 0.017 |
| Isocitric acid_CSF | Metabolome | 0.44 | 0.017 |
| METHYL BETA-D-GALACTOSIDE_Urine | Metabolome | 0.44 | 0.017 |
| L-LYSINE_Ratio | Metabolome | -0.43 | 0.020 |
| Succinylcarnitine_CSF | Metabolome | 0.43 | 0.020 |
| Alpha-aminobutyric acid_CSF | Metabolome | -0.43 | 0.020 |
| N-ACETYL-L-ALANINE_CSF | Metabolome | 0.42 | 0.020 |
| CITRULLINE_Ratio | Metabolome | -0.42 | 0.023 |
| INDOLE-3-ACETAMIDE_Urine | Metabolome | -0.42 | 0.023 |
| HYPOXANTHINE_Ratio | Metabolome | 0.41 | 0.023 |
| Hippuric acid_Serum | Metabolome | 0.41 | 0.024 |
| CREATINE_Ratio | Metabolome | -0.41 | 0.024 |
| PC(40_6)_CSF | Metabolome | 0.40 | 0.032 |
| N-ACETYL-L-ALANINE_Ratio | Metabolome | 0.40 | 0.032 |
| SARCOSINE_CSF | Metabolome | -0.39 | 0.034 |
| L-Carnitine_Ratio | Metabolome | -0.39 | 0.034 |
| CREATINE_CSF | Metabolome | -0.39 | 0.034 |
| N-ACETYLNEURAMINATE_Ratio | Metabolome | 0.39 | 0.034 |
| 5-Oxoproline_CSF | Metabolome | -0.38 | 0.042 |
| GLUCONIC ACID_CSF | Metabolome | 0.38 | 0.042 |
| L-HISTIDINE_CSF | Metabolome | -0.38 | 0.042 |
| L-HISTIDINE_Ratio | Metabolome | -0.38 | 0.042 |
| Butanoylcarnitine_Ratio | Metabolome | -0.38 | 0.043 |
| THEOBROMINE_Ratio | Metabolome | -0.37 | 0.046 |
| N-ACETYLNEURAMINATE_Urine | Metabolome | -0.37 | 0.046 |
| PC(40_8)_Serum | Metabolome | 0.37 | 0.047 |
| N(PAI)-METHYL-L-HISTIDINE_Ratio | Metabolome | -0.37 | 0.048 |
| 5'-METHYLTHIOADENOSINE_Urine | Metabolome | -0.37 | 0.048 |

**Supplementary Table S15: Features associated with QNSE**

| **Overall** |  |  |  |
| --- | --- | --- | --- |
| **Feature** | **Block** | **r** | **FDR** |
| MIF_Ratio | Inflammation | 0.58 | 0.000 |
| SCGF-b_Ratio | Inflammation | 0.45 | 0.004 |
| IFN-g_Ratio | Inflammation | 0.43 | 0.008 |
| MIF_CSF | Inflammation | 0.42 | 0.009 |
| SCGF-b_CSF | Inflammation | 0.40 | 0.011 |
| MIF_Serum | Inflammation | -0.38 | 0.024 |
| PC(37_4)_Serum | Metabolome | 0.47 | 0.015 |
| N-Acetylserine_CSF | Metabolome | 0.47 | 0.015 |
| CITRULLINE_Ratio | Metabolome | -0.47 | 0.015 |
| N-ACETYLNEURAMINATE_Ratio | Metabolome | 0.47 | 0.015 |
| 2-Methylbutyrylglycine_Serum | Metabolome | 0.44 | 0.029 |
| 2-Methylbutyrylglycine_CSF | Metabolome | 0.44 | 0.029 |
| N-ACETYLNEURAMINATE_CSF | Metabolome | 0.43 | 0.029 |
| PC(32_0)_Serum | Metabolome | 0.43 | 0.031 |
| PC(36_4)_Serum | Metabolome | 0.42 | 0.031 |
| SM(30_1)_Serum | Metabolome | 0.42 | 0.031 |

**Supplementary Table S16: Features associated with serum S100B**

| **Overall** |  |  |  |
| --- | --- | --- | --- |
| **Feature** | **Block** | **r** | **FDR** |
| IFN-g_Serum | Inflammation | 0.46 | 0.003 |
| MIF_Ratio | Inflammation | -0.46 | 0.003 |
| IP-10_Serum | Inflammation | 0.43 | 0.005 |
| G-CSF_Ratio | Inflammation | -0.43 | 0.005 |
| MIP-1a_Ratio | Inflammation | -0.43 | 0.005 |
| MIF_Serum | Inflammation | 0.40 | 0.010 |
| PDGF-bb_Ratio | Inflammation | -0.40 | 0.010 |
| MIP-1a_Serum | Inflammation | 0.39 | 0.010 |
| IL-8_Ratio | Inflammation | -0.39 | 0.010 |
| SDF-1a_Serum | Inflammation | 0.37 | 0.019 |
| G-CSF_Serum | Inflammation | 0.36 | 0.021 |
| IFN-g_Ratio | Inflammation | -0.36 | 0.021 |
| IL-16_Ratio | Inflammation | -0.35 | 0.029 |
| Allantoin_Serum | Metabolome | 0.44 | 0.033 |
| PC(30_0)_Serum | Metabolome | -0.46 | 0.033 |
| PC(31_0)_Serum | Metabolome | -0.44 | 0.033 |
| PC(32_2)_Serum | Metabolome | -0.43 | 0.033 |
| Succinylcarnitine_Serum | Metabolome | -0.43 | 0.033 |
| L-TRYPTOPHAN_Ratio | Metabolome | 0.43 | 0.033 |
| Cortisone_Urine | Metabolome | -0.44 | 0.033 |
| Isocitric acid_CSF | Metabolome | -0.42 | 0.036 |
| Alpha-aminobutyric acid_Ratio | Metabolome | 0.42 | 0.036 |
| 2'-DEOXYCYTIDINE 5'-MONOPHOSPHATE_Urine | Metabolome | -0.42 | 0.036 |
| L-CYSTINE_Serum | Metabolome | -0.41 | 0.038 |
| L-ASPARTATE_Serum | Metabolome | 0.40 | 0.041 |
| PC(34_4)_Serum | Metabolome | -0.40 | 0.041 |
| 1,2,3-Propanetricarboxylic_CSF | Metabolome | -0.40 | 0.041 |
| METHYL BETA-D-GALACTOSIDE_Urine | Metabolome | -0.40 | 0.041 |
| N-Alpha-acetyllysine_Serum | Metabolome | -0.40 | 0.042 |
| GUANINE_Urine | Metabolome | -0.40 | 0.042 |

**Supplementary Table S17: Features associated with CSF S100B**

| **Overall** |  |  |  |
| --- | --- | --- | --- |
| **Feature** | **Block** | **r** | **FDR** |
| NFL_CSF | NfL | 0.33 | 0.011 |
| NFL_serum | NfL | 0.27 | 0.022 |
| URIDINE_CSF | Metabolome | -0.50 | 0.016 |
| SM(34_1)_CSF | Metabolome | 0.47 | 0.021 |
| CYTIDINE_CSF | Metabolome | -0.46 | 0.025 |
| Succinic acid_CSF | Metabolome | -0.45 | 0.026 |
| L-METHIONINE_Ratio | Metabolome | 0.44 | 0.026 |
| SM(34_1)_Ratio | Metabolome | 0.44 | 0.026 |
| L-METHIONINE_CSF | Metabolome | 0.42 | 0.033 |
| PC(38_3)_Ratio | Metabolome | 0.42 | 0.033 |
| SM(42_2)_Ratio | Metabolome | 0.42 | 0.033 |
| SM(42_2)_CSF | Metabolome | 0.42 | 0.033 |
| N-ACETYL-L-PHENYLALANINE_Serum | Metabolome | 0.41 | 0.042 |
| SM(36_2)_CSF | Metabolome | 0.40 | 0.047 |

**Supplementary Table S18: Features associated with QS100B**

| **Overall** |  |  |  |
| --- | --- | --- | --- |
| **Feature** | **Block** | **r** | **FDR** |
| MIF_Ratio |  | 0.51 | <0.001 |
| MIP-1a_Ratio |  | 0.48 | 0.001 |
| IL-8_Ratio |  | 0.43 | 0.006 |
| IFN-g_Serum |  | -0.41 | 0.010 |
| IFN-g_Ratio |  | 0.41 | 0.010 |
| SDF-1a_Serum |  | -0.39 | 0.013 |
| MIF_Serum |  | -0.38 | 0.017 |
| MIP-1a_CSF |  | 0.37 | 0.023 |
| PDGF-bb_Ratio |  | 0.37 | 0.023 |
| G-CSF_Ratio |  | 0.36 | 0.027 |
| IL-8_CSF |  | 0.34 | 0.035 |
| MIF_CSF |  | 0.34 | 0.035 |
| IP-10_Ratio |  | 0.34 | 0.035 |
| IP-10_Serum |  | -0.33 | 0.043 |
| IL-16_Ratio |  | 0.33 | 0.043 |
| IL-8_Serum |  | -0.32 | 0.047 |
